# A hybrid yang transform adomian decomposition method for solving time-fractional nonlinear partial differential equation

**DOI:** 10.1186/s13104-024-06877-7

**Published:** 2024-08-16

**Authors:** Alemu Senbeta Bekela, Alemayehu Tamirie Deresse

**Affiliations:** 1 Department of Mathematics, Samara University, Samara, Ethiopia; 2 Department of Mathematics, Faculty of Natural Sciences, Mizan Tepi University, Tepi, Ethiopia

**Keywords:** Caputo fractional derivative, Time-fractional nonlinear partial differential equations, Yang transform, Adomian decomposition method

## Abstract

Nonlinear time-fractional partial differential equations (NTFPDEs) play a great role in the mathematical modeling of real-world phenomena like traffic models, the design of earthquakes, fractional stochastic systems, diffusion processes, and control processing. Solving such problems is reasonably challenging, and the nonlinear part and fractional operator make them more problematic. Thus, developing suitable numerical methods is an active area of research. In this paper, we develop a new numerical method called Yang transform Adomian decomposition method (YTADM) by mixing the Yang transform and the Adomian decomposition method for solving NTFPDEs. The derivative of the problem is considered in sense of Caputo fractional order. The stability and convergence of the developed method are discussed in the Banach space sense. The effectiveness, validity, and practicability of the method are demonstrated by solving four examples of NTFPEs. The findings suggest that the proposed method gives a better solution than other compared numerical methods. Additionally, the proposed scheme achieves an accurate solution with a few numbers of iteration, and thus the method is suitable for handling a wide class of NTFPDEs arising in the application of nonlinear phenomena.

## Introduction

Different natural phenomena that appear in various areas of science and engineering can be successfully demonstrated by using the notion of fractional calculus [1]. Fractional calculus plays a great role in the mathematical modeling of real-world phenomena, for example, reaction–diffusion processes [2, 3], decentralized wireless networks [4, 5], water wave movement [6, 7], signal processing [8, 9], population growth [10, 11], design of earthquake [12, 13], traffic models with fractional derivatives [14], diffusion processes [15], fractional stochastic systems [16–19], control processing [20, 21], medical sciences [22] and many other physical processes [23–26].

In most cases, fractional differential equations (FDEs) are considered a generalization of differential equations (DEs) because they describe functional values at every continuous point without losing any memory or hereditary behaviors of natural phenomena. For this reason, many authors have studied FDEs for modeling and deep understanding of real-world natural phenomena such as business models with fractional derivations, evaluation of viscoelastic surface characteristics, human behavior in the field of mathematical psychology [27, 28], models of love between humans [29], and models of happiness and in other fields of applications [23–25, 30, 31].

Nonlinear time-fractional partial differential equations (NTFPDEs) have played an alternative role in diverse fields such as applied science, mathematics, physics, medicine, biology, geology, chemistry, and engineering due to the non-locality nature of the power-law structure with arbitrary positive order. In the modern age, it is impossible to model many real-world problems without using nonlinear FPDEs. Fractional calculus can be called this century’s calculus because of the diversity of applications in every discipline of applied science, technology, and engineering [32–34].

In applied mathematics and many other areas of science, it is important to obtain accurate solutions to FPDEs. In general, FPDEs do not have closed-form exact solutions for most problems, and it is very difficult to obtain exact solutions [35]. For this reason, various numerical methods have been developed for solving linear and nonlinear FPDEs. For instance, Mohamed and Torky [33] proposed the Legendre multiwavelet Galerkin method to solve the conformable fractional modified KdV–ZK equation. Singh et al*.* [36] announced the homotopy perturbation Sumudu transform method for nonlinear FPDEs arising in the spatial diffusion of biological populations. Wang and Liu [37] familiarized themselves with the new Sumudu transform iterative method for the time‑fractional Cauchy reaction–diffusion equation. Ziane et al. [38] introduced the Elzaki transform combined with the variational iteration method for PDEs of fractional order. Dehestani et al. [39] proposed Legendre–Laguerre functions based on Legendre and Laguerre polynomials for solving a class of linear and nonlinear time–space fractional PDEs with variable coefficients. Dehestani et al. [40] presents the discrete scheme based on Genocchi polynomials and fractional Laguerre functions to solve multiterm variable-order time-fractional PDEs in the large interval. Dehestani et al. [41] familiarized themselves with the fractional-order Genocchi–Petrov–Galerkin method to investigate the approximate solution of time–space fractional Fokker–Planck equations. Wang et al. [42] proposed two analytical methods, the residual power series method and the homotopy analysis transform method, to solve NTFPDEs with proportional delay.

Recently, researchers have used several numerical methods to solve different types of NTFPDEs. For example, Bekela et al. [32] applied the hybrid numerical method of Laplace-like transform and variational theory for solving NTFPDEs with proportional delay. Malyk et al*.* [43] employed the Yang–Abdel–Cattani derivative operator for solving the nonlinear fractional diffusion equation. The numerical method is presented by using a novel spline technique for solving fourth-order time-fractional evolution problems [44]. Al-Deiakeh et al*.* [45] introduced an approximation for nonlinear FPDE using the combination of the multi-Laplace transform and ADM. Mallick et al. [46] proposed an iterative method for solving time-fractional PDEs with proportional delays. A wide application of NTFPDEs in many real life phenomena and the growing of to search for suitable numerical methods motivate us to propose a hybrid numerical method called the Yang transform Adomian decomposition method (YTADM) for solving NTFPDEs.

George Adomian introduced a modern mathematical method to solve nonlinear DEs in the 1980s, described as ADM [47–49]. Similarly, another powerful method found by Yang to solve linear PDEs was described as YT, which transforms the initial DEs into an algebraic equation [50–52]. However, it does not handle nonlinear terms in NTFPDEs because of the difficulties that are caused by nonlinear terms. ADM is a powerful numerical method for solving nonlinear DEs, but it does not properly decompose the fractional order parts. The main objective of this study is to combine two methods for solving the NTFPDEs: YT is used to decompose FDEs into algebraic equations, and ADM is used to decompose the nonlinear terms in NTFPDEs into a series solution. The stability and convergence conditions of the proposed method were analyzed. In addition, some test examples of NTFPDEs are solved to illustrate the capability, accuracy, and simplicity of the proposed method.

Furthermore, to demonstrate the versatility and robustness of the YTADM in different areas of science, we take four examples that have a wide range of applications in various fields of science and technology. Accordingly, first we considered the nonlinear time-fractional Newell-Whitehead-Segel equation (NWSE), which predicts the existence of traveling wave patterns. These kinds of systems can be observed in a variety of natural systems, including physical, biological, and chemical systems. For example, in medicine: the mechanisms of spreading epidemic diseases, healing wounded tissue, chemical reactions of multiple substances, and neutron diffusion theory all belong to reaction–diffusion systems [49].

Then, we considered the nonlinear fractional Cauchy reaction–diffusion equation (CRDE), which is used to describe the evolution of a system over time with reaction and diffusion processes in various fields. For instance, in biology and medicine, these equations are used to model the spreading of diseases, tumor growth, etc. In chemistry, CRDEs are employed to understand chemical reactions that exhibit complex kinetics and diffusion processes. In population dynamics, these equations are employed to model the behavior of ecological and natural populations. In material science, CRDEs are expended for modeling transport marvels in porous media whereas in finance and economics, they are used for modeling the financial markets with memory goods and long-range dependence [47, 48], and references therein.

Hereafter, we solve the nonlinear time-fractional Fornberg-Whitham equation (FWE). The FWE is a type of traveling wave equation and have variety of applications in physics and engineering. Such as in the propagation of electrical signals and optimization of guided communication systems. In mathematical physics to investigate how non-linear dispersive water waves break [50]. Final, we considered one problem from nonlinear time-fractional Fokker–Planck equation (FPE). The nonlinear time-fractional FPE arises in various fields like in chemistry, natural science, including astrophysics problems, biological applications, chemical physics, and other fields [51].

Here, the numerical results obtained by YTADM are summarized in tables and figures to illustrate easily understanding for various fractional order $\alpha$. For the fractional order $\alpha =1$, the obtained numerical solution have excellent agreement with the exact solution. It is evident from the theory of fractional calculus that the approximate solution of the problem with derivative $1$ continuously tends to the exact solution when the fractional derivative $\alpha (m-1<\alpha \le m)$ tends to positive integer $m=1$[52].

The remaining paper is organized in the following way: Sect. "Preliminaries" of this work gives detailed concepts related to fractional integrals, derivatives, and YT. Sect. "A hybrid numerical method for solving NTFPDEs" familiarizes reader with the mathematical formulation of YTADM using YT and ADM. Sects. "Stability analysis of YTADM to solve NTFPDEs" and "Convergence analysis of YTADM for solving NTFPDEs" offer stability and convergence of the proposed method in the Banach space sense. Sect. "Numerical results and discussions" presents a numerical simulation of the method on some NTFPDEs. Finally, the conclusion of the method is presented in Sect. "Conclusion".

## Preliminaries

This part is devoted to some basic concepts and definitions of YT, fractional integrals, and fractional derivatives, which are essential for accepting the remainder of the monograph.

### Definition 2.1

[6, 7]. A real function $f\left(t\right), t>0$ is in the space ${C}_{\tau }, \tau \in R$ if there exists a real number $\left(p>\tau \right)$, such that $f\left(t\right)={t}^{p}{f}_{1}\left(t\right)$, where ${f}_{1}\left(t\right)\in C\left[0,\infty )\right.$, and it is said to be in the space ${C}_{\tau }^{n}$ if ${f}^{\left(n\right)}\in {C}_{\tau }, n\in N$, clearly ${C}_{\tau }\subset {C}_{\rho }$ if $\rho \le \tau$.

### Definition 2.2

[24, 25]. The left sides Riemann–Liouville fractional integral of order $\alpha \ge 0$, of a function $f\left(t\right)\in {C}_{\tau },\tau \ge -1$ is defined as.

$$D_{{a,t}}^{{ - \alpha }} f\left( t \right)\, = \,\left\{ \begin{gathered} \frac{1}{{\Gamma \left( \alpha \right)}}\smallint _{a}^{t} \left( {t - \mu } \right)^{{\alpha - 1}} f\left( \mu \right)d\mu ,\alpha \, < 0,\quad t > 0, \hfill \\ f\left( t \right),\quad \alpha = 0 \hfill \\ \end{gathered} \right.$$

where $\Gamma \left(.\right)$ is the gamma function.

### Definition 2.3

[7, 55]. The fractional derivative of $f(t)$ in the Caputo sense is defined as.

$${{}_{a}{}^{C}D}_{t}^{\alpha }f\left(t\right)=\frac{1}{\Gamma \left(n-\alpha \right)}{\int }_{a}^{t}{\left(t-\mu \right)}^{n-\left(\alpha +1\right)}{f}^{\left(n\right)}\left(\mu \right)d\mu ,$$

where ${{}_{a}{}^{C}D}_{t}^{\alpha }$ is the Caputo fractional derivative operator of order $\alpha$, $n-1<\alpha \le n, n\in N$.

### Definition 2.4

[56, 57]. The YT is defined over the set of function.

$$A=\left\{f\left(t\right):\exists C,{m}_{1,}{m}_{2}>0,\left|f(t)\right|<C{e}^{\frac{\left|t\right|}{{m}_{j}}}, if t\in {(-1)}^{j}\times [0,\infty ), j=\text{1,2}\right\},$$

where $C$ is constant and ${m}_{1},{m}_{2}$ are either finite or infinite by the following integral formula

$$Y\left[f(t)\right]=T\left(v\right)={\int }_{0}^{\infty }{e}^{\frac{-t}{v}}f\left(t\right)dt, v>0.$$

It converges if the limit of the integral exists, and diverges if not. Accordingly, the following results follows directly from the above integral formula of YT [56, 59]:

1. $$Y\left[1\right]=v$$,

2. $$Y\left[t\right]={v}^{2}$$,

3. $$Y\left[{t}^{n}\right]=n!{v}^{n+1}=\Gamma \left(n+1\right){v}^{n+1}, n\in N,$$

4. $$Y\left[{t}^{\alpha }\right]=\Gamma \left(\alpha +1\right){v}^{\alpha +1}, \alpha >0,$$

5. $$Y\left[af\left(t\right)+bg\left(t\right)\right]=aY\left[f\left(t\right)\right]+bY\left[g\left(t\right)\right],$$ linearity property.

### Definition 2.5

[56, 59]. The YT of the ${n}^{th}$ derivative of the function $f\left(t\right)$ is defined as

$$Y\left[{f}^{n}\left(t\right)\right]=\frac{T\left(v\right)}{{v}^{n}}-\sum_{k=0}^{n-1}\frac{{f}^{\left(k\right)}\left(0\right)}{{v}^{n-\left(k+1\right)}}.$$

### Definition 2.6

[58]. The YT of the function $f\left(t\right)$ in the Caputo fractional derivative is defined as.

$$Y\left[{}_{0}{}^{C}{D}_{t}^{\alpha }f\left(t\right)\right]=\frac{T\left(v\right)}{{v}^{\alpha }}-\sum_{k=0}^{n-1}\frac{{f}^{\left(k\right)}\left(0\right)}{{v}^{\alpha -\left(k+1\right)}},$$

for $$T\left(v\right)=Y\left[f\left(t\right)\right]$$ and $$n-1<\alpha \le n, n\in N$$.

### Definition 2.7

([32]) Let $P:X\to X$ be a mapping of a set $X$ into itself. The fixed point of $P$ is $x\in X$ which is mapped onto itself, that is,

$$Px= x,$$

the image $Px$ coincides with $x$.

### Definition 2.8

(Contraction [32]) Let $X=(X, d)$ be a metric space. A mapping $P:X\to X$ is called a contraction on $X$ if there is $0\le \varepsilon <1$ such that, for all $x,y\in X$,

$$d\left(Px,Py\right)\le \varepsilon d\left(x,y\right).$$

### Theorem 2.1

(Banach’s fixed point theorem [32]) Consider a metric space $X=(X, d)$*,* where $x\ne \varnothing$*.* Suppose that $X$ is complete, and let $P:X\to X$ be a contraction on $X$*.* Then $P$ has a unique fixed point*.*

### Theorem 2.2

[60]. Let $\left(X,\Vert .\Vert \right)$ be a Banach space and $P$ be a self-map of $X$* (*$P:X\to X$*),* satisfying.

$$\left\| {P_{x} - P_{y} } \right\| \le C\left\| {x - P_{x} } \right\| + \varepsilon \left\| {x - y} \right\|,\,\forall x,y \in X, C \ge 0, 0 < \varepsilon < 1,$$

then $P$ is Picard, $\text{P}$-stable.

## A hybrid numerical method for solving NTFPDEs

In this section, we derive a hybrid numerical method that uses YT and ADM. We named this developed method YTADM. To illustrate the basic idea of this method, we consider the following general NTFPDEs with a source term [38, 58]

1 $$_{0}^{C} D_{t}^{\alpha } u\left( {x,t} \right) + Ru\left( {x,t} \right) + Nu\left( {x,t} \right) = f\left( {x,t} \right),\alpha \in \left[ {0,1} \right],$$

with the initial condition

2 $$u\left(x,0\right)=g\left(x\right),$$

where ${}_{0}{}^{C}{D}_{t}^{\alpha }=\frac{{\partial }^{\alpha }}{\partial {t}^{\alpha }}$ is the Caputo fractional derivative of order $\alpha$ with respect to $t$, $R$ is the linear differential operator, $N$ represents the general nonlinear differential operator and $f\left(x,t\right)$ is the source term. To develop YTADM for solving (1) and (2), we follow the following steps.

**Step 1:** Apply YT on both sides of (1) to obtain

3 $$Y\left[{}_{0}{}^{C}{D}_{t}^{\alpha }u\left(x,t\right)\right]+Y\left[Ru\left(x,t\right)\right]+Y\left[Nu\left(x,t\right)\right]=Y\left[f\left(x,t\right)\right].$$

By using Definition 2.6 we transform the fractional derivative on the left side of (3) in to

4 $$\frac{Y\left[u\left(x,t\right)\right]}{{v}^{\alpha }}-\sum_{k=0}^{n-1}{v}^{k-\alpha +1}{u}^{\left(k\right)}\left(x,0\right)=Y\left[f\left(x,t\right)-Ru\left(x,t\right)-Nu\left(x,t\right)\right].$$

Note that in this step, YT changes the fractional derivative into an algebraic equation, and using the given initial condition, we can rewrite (4) as

$$\frac{T\left(x,v\right)}{{v}^{\alpha }}={v}^{1-\alpha }u\left(x,0\right)+Y\left[f\left(x,t\right)-Ru\left(x,t\right)-Nu\left(x,t\right)\right],$$

$$T\left(x,v\right)=vu\left(x,0\right)+{v}^{\alpha }Y\left[f\left(x,t\right)-Ru\left(x,t\right)-Nu\left(x,t\right)\right],$$

where $$T\left(x,v\right)=Y\left[u\left(x,t\right)\right]$$.

By the linearity property of the YT, the above equation becomes

5 $$T\left(x,v\right)=vu\left(x,0\right)+{v}^{\alpha }Y\left[f\left(x,t\right)\right]-{v}^{\alpha }Y\left[Ru\left(x,t\right)+Nu\left(x,t\right)\right].$$

**Step 2:** Apply the inverse YT on both sides of (5), to obtain

6 $$u\left(x,t\right)\ , =\, u\left(x,0\right)+{Y}^{-1}\left[{v}^{\alpha }Y\left[f\left(x,t\right)\right]\right]-{Y}^{-1}\left[{v}^{\alpha }Y\left[Ru\left(x,t\right)+Nu\left(x,t\right)\right]\right],$$

where $$u\left(x,t\right)=\,{Y}^{-1}\left[T\left(x,v\right)\right].$$

**Step 3:** In order to decompose the nonlinear term in NTFPDE and generate a series solution of the proposed method, the ADM is applied. The ADM defines the solution in an infinite series of linear terms as

7 $$u\left(x,t\right)=\sum_{m=0}^{\infty }{u}_{m}\left(x,t\right),$$

and the nonlinear term in the problem expressed in terms of the Adomian polynomial as follows:

8 $$Nu\left(x,t\right)=\sum_{m=0}^{\infty }{A}_{n}\left(u\right),$$

where

9 $${A}_{m}=\frac{1}{m!}{\frac{{d}^{m}}{d{\lambda }^{m}}\left[N\left(\sum_{i=0}^{m}{\lambda }^{i}{u}_{i}\right)\right]}_{\lambda =0},m=\text{1,2},\dots$$

**Step 4:** Substituting the obtained values in (7) and (8) into (6), we obtain

10 $$\sum_{m=0}^{\infty }{u}_{m}\left(x,t\right)=u\left(x,0\right)+{Y}^{-1}\left[{v}^{\alpha }\left(Y\left[f\left(x,t\right)\right]\right)\right]-{Y}^{-1}\left[{v}^{\alpha }Y\left[\sum_{m=0}^{\infty }R{u}_{m}\left(x,t\right)+\sum_{m=0}^{\infty }{A}_{m}\left(u\right)\right]\right].$$

**Step 5:** To obtain the component solutions of the proposed method, we equating the terms on both sides of (10), and get the following relation

$${u}_{0}\left(x,t\right)=u\left(x,0\right),$$

$${u}_{1}\left(x,t\right)={Y}^{-1}\left[{v}^{\alpha }\left(Y\left[f\left(x,t\right)\right]\right)\right]-{Y}^{-1}\left[{v}^{\alpha }Y\left[R{u}_{0}\left(x,t\right)+{A}_{0}\left(u\right)\right]\right],$$

11 $${u}_{m+1}\left(x,t\right)=-{Y}^{-1}\left[{v}^{\alpha }Y\left[R{u}_{m}\left(x,t\right)+{A}_{m}\left(u\right)\right]\right], m\ge 1.$$

**Step 6:** Following component approximate solution, we obtain the general solution in a series form as

12 $$u\left(x,t\right)=\sum_{m=0}^{\infty }{u}_{m}\left(x,t\right)={u}_{0}\left(x,t\right)+{u}_{1}\left(x,t\right)+{u}_{2}\left(x,t\right)+\dots .$$

Note that it is impossible to find infinite component of solution. Hence, after ${\left(m+1\right)}^{th}$ truncation, the iteration formula of YTADM is given by

13 $${u}_{m+1}\left(x,t\right)=u\left(x,0\right)+{Y}^{-1}\left[{v}^{\alpha }Y\left[f\left(x,t\right)-R{u}_{m}\left(x,t\right)-{A}_{m}\left(u\right)\right]\right],$$

which is the ${\left(m+1\right)}^{th}$ approximate solution of the NTFPDE in (1) for $0<\alpha \le 1$.

### Stability analysis of YTADM to solve NTFPDEs

In this section, we discuss the stability analysis of the method presented in Sect. "A hybrid numerical method for solving NTFPDEs". For this, we state and prove an essential condition concerning the stability of YTADM when it is applied to solve NTFPDEs. To reveal Picard stability, it enough to show that the mapping associated with YTADM in (13) satisfies the conditions of Theorem 2.2.

#### Theorem 3.1

Let $\left(\text{X},\Vert .\Vert \right)$ be a Banach space and $P:X\to X$ be a self-map of $X$. Then, the iteration procedure of YTADM defined by.

$$P\left({u}_{m}(x,t)\right) =\, {u}_{m+1}\left(x,t\right)=u\left(x,0\right)+{Y}^{-1}\left[{v}^{\alpha }Y\left[f\left(x,t\right)-R{u}_{m}\left(x,t\right)-{A}_{m}\left(u\right)\right]\right],$$

is P-stable if.

i) $$\Vert R{u}_{m}\left(x,t\right)-R{u}_{n}\left(x,t\right)\Vert \le {\varepsilon }_{0}\Vert {u}_{m}\left(x,t\right)-{u}_{n}\left(x,t\right)\Vert$$ for some $${\varepsilon }_{0}\in {R}^{+}$$,

ii) $\Vert {A}_{m}\left(u\right)-{A}_{n}\left(u\right)\Vert \le {\varepsilon }_{1}\Vert {u}_{m}\left(x,t\right)-{u}_{n}\left(x,t\right)\Vert$ for some ${\varepsilon }_{1}\in {R}^{+}$,

iii) $$\varepsilon =\left({\varepsilon }_{0}+{\varepsilon }_{1}\right)\Vert \frac{{t}^{\alpha }}{\Gamma \left(\alpha +1\right)}\Vert <1$$.

#### Proof.

First we show that $P$ has a fixed point. To do this, for $n,m\in N$, we have.

14 $$P\left({u}_{n}(x,t)\right)=u\left(x,0\right)+{Y}^{-1}\left[{v}^{\alpha }Y\left[f\left(x,t\right)-R{u}_{n}\left(x,t\right)-{A}_{n}\left(u\right)\right]\right],$$

15 $$P\left({u}_{m}(x,t)\right)=u\left(x,0\right)+{Y}^{-1}\left[{v}^{\alpha }Y\left[f\left(x,t\right)-R{u}_{m}\left(x,t\right)-{A}_{m}\left(u\right)\right]\right].$$

By subtracting (15) from (14), we obtain

16 $$P\left({u}_{n}\left(x,t\right)\right)-P\left({u}_{m}\left(x,t\right)\right)={Y}^{-1}\left[{v}^{\alpha }Y\left[R{u}_{m}\left(x,t\right)+{A}_{m}\left(u\right)\right]\right]-{Y}^{-1}\left[{v}^{\alpha }Y\left[R{u}_{n}\left(x,t\right)+{A}_{n}\left(u\right)\right]\right].$$

Taking the norm on both sides of (16), without loss of generality, we have

$$\left\| {P\left( {u_{n} (x,t)} \right) - P\left( {u_{m} \left( {x,t} \right)} \right)} \right\| = \left\| {\begin{array}{*{20}c} {Y^{{ - 1}} \left[ {v^{\alpha } Y\left[ {Ru_{m} \left( {x,t} \right) + A_{m} \left( u \right)} \right]} \right]} \\ { - Y^{{ - 1}} \left[ {v^{\alpha } Y\left[ {Ru_{n} \left( {x,t} \right) + A_{n} \left( u \right)} \right]} \right]} \\ \end{array} } \right\|.$$

Using the linearity property of the YT and its inverse, we obtain.

$$\left\| {P\left( {u_{n} (x,t)} \right) - P\left( {u_{m} \left( {x,t} \right)} \right)} \right\| = \left\| {\begin{array}{*{20}c} {Y^{{ - 1}} \left[ {v^{\alpha } Y\left[ {Ru_{m} \left( {x,t} \right)} \right]} \right] + Y^{{ - 1}} \left[ {v^{\alpha } Y\left[ {A_{m} \left( u \right)} \right]} \right]} \\ { - Y^{{ - 1}} \left[ {v^{\alpha } Y\left[ {Ru_{n} \left( {x,t} \right)} \right]} \right] - Y^{{ - 1}} \left[ {v^{\alpha } Y\left[ {A_{n} \left( u \right)} \right]} \right]} \\ \end{array} } \right\|.$$

Using the properties of the norm, we proceed.

17 $$\Vert P\left({u}_{n}\left(x,t\right)\right)-P\left({u}_{m}\left(x,t\right)\right)\Vert \le \Vert {Y}^{-1}\left[{v}^{\alpha }Y\left[R{u}_{m}\left(x,t\right)-R{u}_{n}\left(x,t\right)\right]\right]\Vert +\Vert {Y}^{-1}\left[{v}^{\alpha }Y\left[{A}_{m}\left(u\right)-{A}_{n}\left(u\right)\right]\right]\Vert .$$

Now, assuming that.

$$\Vert R{u}_{m}\left(x,t\right)-R{u}_{n}\left(x,t\right)\Vert \le {\varepsilon }_{0}\Vert {u}_{m}\left(x,t\right)-{u}_{n}\left(x,t\right)\Vert$$ and.

$$\Vert {A}_{m}\left(u\right)-{A}_{n}\left(u\right)\Vert \le {\varepsilon }_{1}\Vert {u}_{m}\left(x,t\right)-{u}_{n}\left(x,t\right)\Vert$$ for some $${\varepsilon }_{0},{\varepsilon }_{1}\in {R}^{+}$$.

Then, (17) becomes

18 $$\Vert P\left({u}_{n}(x,t)\right)-P\left({u}_{m}\left(x,t\right)\right)\Vert \le \left(\begin{array}{c}{\varepsilon }_{0}\Vert {u}_{m}\left(x,t\right)-{u}_{n}\left(x,t\right)\Vert \\ +{\varepsilon }_{1}\Vert {u}_{m}\left(x,t\right)-{u}_{n}\left(x,t\right)\Vert \end{array}\right)\Vert {Y}^{-1}\left[{v}^{\alpha }Y\left[1\right]\right]\Vert .$$

However, from the properties of YT, we have.

$$\left\| {Y^{{ - 1}} \left[ {v^{\alpha } Y\left[ 1 \right]} \right]} \right\| = \left\| {Y^{{ - 1}} \left[ {v^{\alpha } \left( v \right)} \right]} \right\| = \left\| {Y^{{ - 1}} \left[ {v^{{\alpha + 1}} } \right]} \right\| = \left\| {\frac{{t^{\alpha } }}{{\Gamma \left( {\alpha + 1} \right)}}} \right\|.$$

Therefore, from (18), we obtain

19 $$\begin{aligned} \left\| {P(u_{n} (x,t)) - P(u_{m} (x,t))} \right\| \le & \left( {\begin{array}{*{20}c} {\varepsilon _{0} \left\| {u_{m} \left( {x,t} \right) - u_{n} \left( {x,t} \right)} \right\|} \\ { + \varepsilon _{1} \left\| {u_{m} \left( {x,t} \right) - \left( {x,t} \right)} \right\|} \\ \end{array} } \right)\left\| {\frac{{t^{\alpha } }}{{\Gamma \left( {\alpha + 1} \right)}}} \right\|, \\ \le & \left( {\varepsilon _{0} + \varepsilon _{1} } \right)\left\| {\frac{{t^{\alpha } }}{{\Gamma \left( {\alpha + 1} \right)}}} \right\|\left\| {u_{n} \left( {x,t} \right) - u_{m} \left( {x,t} \right)} \right\|, \\ \le & \varepsilon \left\| {u_{n} \left( {x,t} \right) - u_{m} \left( {x,t} \right)} \right\|, \\ \end{aligned}$$

where $$\varepsilon = \left( {\varepsilon _{0} + \varepsilon _{1} } \right)\,\left\| {\frac{{t^{\alpha } }}{{\Gamma \left( {\alpha + 1} \right)}}} \right\|$$.

Hence, the self-mapping $P$ has a fixed point. Now, we show $P$ satisfies the condition in Theorem $3.1$. For this, we have

$$\Vert P\left({u}_{n}(x,t)\right)-P\left({u}_{m}\left(x,t\right)\right)\Vert \le C\Vert {u}_{n}\left(x,t\right)-P\left({u}_{m}\left(x,t\right)\right)\Vert +\varepsilon \Vert {v}_{n}\left(x,t\right)-{v}_{m}\left(x,t\right)\Vert ,$$

for $C = 0,\varepsilon = \left( {\varepsilon _{0} + \varepsilon _{1} } \right)\,\left\| {\frac{{t^{\alpha } }}{{\Gamma \left( {\alpha + 1} \right)}}} \right\|{\text{ < }}\,1.$ This shows that the conditions of Theorem 3.1 hold for self-mapping $P$. Hence, by Theorem 2.2, YTADM is Picard *T*-stable if $\varepsilon <1$.

### Convergence analysis of YTADM for solving NTFPDEs

In this section, we prove the convergence of YTADM when applied to NTFPDEs.

#### Theorem 3.2.

Let $\left(X,\Vert .\Vert \right)$ be a Banach space and $P: X\to X$ be a mapping associated with YTADM be defined by (13). Then, $P$ has a unique fixed point and the sequence solution ${\left\{{u}_{n}\left(x,t\right)\right\}}_{n=0}^{\infty }$ converges to the fixed point of $X$ with an initial value ${u}_{0}\in X$, if $0<\varepsilon <1$ such that $\Vert {u}_{n+1}\left(x,t\right)\Vert \le \varepsilon \Vert {u}_{n}\left(x,t\right)\Vert$.

#### Proof.

First, we show the existence of a fixed point of $P$. For this define that $\left\{{s}_{n}\right\}$ is the sequence of partial sums of the series Eq. (13) as,

$${s}_{0}={u}_{0}\left(x,t\right), {s}_{1}={u}_{0}\left(x,t\right)+{u}_{1}\left(x,t\right), {s}_{2}={u}_{0}\left(x,t\right)+{u}_{1}\left(x,t\right)+{u}_{2}\left(x,t\right),\dots ,$$

$${s}_{n}={u}_{0}\left(x,t\right)+{u}_{1}\left(x,t\right)+{u}_{2}\left(x,t\right)+\dots +{u}_{n}\left(x,t\right).$$

Now, we show that ${\left\{{s}_{n}\right\}}_{n=0}^{\infty }$ is a Cauchy sequence in Banach space $\left(X,\Vert .\Vert \right)$. For this purpose, we consider.

20 $$\Vert {s}_{n+1}- {s}_{n}\Vert =\Vert {u}_{n+1}\left(x,t\right)\Vert \le \varepsilon \Vert {u}_{n}\left(x,t\right)\Vert \le {\varepsilon }^{2}\Vert {u}_{n-1}\left(x,t\right)\Vert \le \dots \le {\varepsilon }^{n+1}\Vert {u}_{0}\left(x,t\right)\Vert .$$

Now, for every $m,n\in N$ with $n>m$, then by using (20) and triangle inequality, we obtain

21 $$\begin{aligned} \left\| {s_{n} - s_{m} } \right\| =\, & \left\| {\left( {s_{n} - s_{{n - 1}} } \right) + \left( {s_{{n - 1}} - s_{{n - 2}} } \right) + \ldots + \left( {s_{{m + 1}} - s_{m} } \right)} \right\|, \\ \le & \left\| {s_{n} - s_{{n - 1}} } \right\| + \left\| {s_{{n - 1}} - s_{{n - 2}} } \right\| + \ldots + \left\| {s_{{m + 1}} - s_{m} } \right\|, \\ \le & \varepsilon ^{n} \left\| {u_{0} \left( {x,t} \right)} \right\| + \varepsilon ^{{n - 1}} \left\| {u_{0} \left( {x,t} \right)} \right\| + \ldots + \varepsilon ^{{m + 1}} \left\| {u_{0} \left( {x,t} \right)} \right\|, \\ \le & \varepsilon ^{{m + 1}} \left\| {u_{0} \left( {x,t} \right)} \right\|\left( {1 + \varepsilon + \varepsilon ^{2} + \ldots + \varepsilon ^{{n - m}} } \right). \\ \end{aligned}$$

Since $0<\varepsilon <1$, the sum $1+\varepsilon +{\varepsilon }^{2}+\dots +{\varepsilon }^{n-m}$ represents a finite geometric sequence whose total sum is $\frac{1-{\varepsilon }^{n-m}}{1-\varepsilon }$. Now, we have $1-{\varepsilon }^{n-m}<1$; then, (21) becomes.

22 $$\Vert {s}_{n}- {s}_{m}\Vert \le \frac{{\varepsilon }^{m+1}}{1-\varepsilon }\Vert {u}_{0}\left(x,t\right)\Vert .$$

Since ${u}_{0}\left(x,t\right)$ is bounded,

$$\underset{n,m\to \infty }{\text{lim}}\Vert {s}_{n}- {s}_{m}\Vert =0.$$

This implies that ${s}_{n}={s}_{m}$. Therefore, ${\left\{{s}_{n}\right\}}_{n=0}^{\infty }$ is a Cauchy sequence and is convergent. Hence, $P$ has a fixed point.

Next, to complete this proof, let $\left\{{u}_{n}\left(x,t\right)\right\}$ converge to $u\in X$ and we need to show that $u$ is the unique fixed point of $P$. To achieve this, let $l$ be another fixed point of $P$. Then, by (19), we have

$$\begin{gathered} \left\| {u - l} \right\| = \left\| {Pu - Pl} \right\| \le \varepsilon \left\| {u - l} \right\|, \hfill \\ \left\| {u - l} \right\| \le \varepsilon \left\| {u - l} \right\| \Rightarrow \left( {1 - \varepsilon } \right)\left\| {u - l} \right\| \le 0. \hfill \\ \end{gathered}$$

Since,$\left(1-\varepsilon \right)<0$ for $0<\varepsilon <1$, the above inequality can only hold if $\Vert u-l\Vert =0$ implies $u=l$. Hence, $u$ is the unique fixed point of $P$, which completes the proof.

## Numerical results and discussions

In this section, four examples of NTFPDEs are solved by using the YTADM. The validity of the proposed numerical method is observed in terms of its absolute errors. To show the behaviors of the corresponding examples, some figures are also plotted for fractional orders $\alpha$. All the results are calculated using MATLAB.

### ***Example 4.1***

[61]. Consider the following nonlinear time-fractional Newell-Whitehead-Segel equation.

23 $$\frac{{\partial }^{\alpha }}{\partial {t}^{\alpha }}u\left(x,t\right)=\frac{{\partial }^{2}}{\partial {x}^{2}}u\left(x,t\right)+2u\left(x,t\right)-3{u}^{2}\left(x,t\right), 0<\alpha \le 1,$$

with initial conditions

$$u\left(x,0\right)=\lambda .$$

The exact solution of this problem is $u\left(x,t\right)=\frac{-\frac{2}{3}\lambda {e}^{2t}}{-\frac{2}{3}+\lambda -\lambda {e}^{2t}}.$for the special case $\alpha =1$. For simplicity, we drop $\left(x,t\right)$ from $u\left(x,t\right)$ and $\frac{{\partial }^{2}}{\partial {x}^{2}}u\left(x,t\right).$

**Step 1:** Applying YT on both sides of (23) and using the differentiation property, we have

24 $$\frac{T\left(x,v\right)}{{v}^{\alpha }}-\sum_{k=0}^{m-1}{v}^{k-\alpha +1}{u}^{\left(k\right)}\left(x,0\right)=Y\left[\frac{{\partial }^{2}}{\partial {x}^{2}}u+2u-3{u}^{2}\right].$$

In our case $0<\alpha \le 1$, (24) is reduced to

$$\frac{1}{{v}^{\alpha }}T\left(x,v\right)-{v}^{\alpha -1}u\left(x,0\right)=Y\left[\frac{{\partial }^{2}}{\partial {x}^{2}}u+2u-3{u}^{2}\right].$$

Using the given initial condition, we obtain

25 $$T\left(x,v\right)=\lambda v+{v}^{\alpha }Y\left[\frac{{\partial }^{2}}{\partial {x}^{2}}u+2u-3{u}^{2}\right].$$

**Step 2:** Applying the inverse YT on both sides of (25), we obtain.

$$u\left(x,t\right)=\lambda {Y}^{-1}\left[v\right]+{Y}^{-1}\left[{v}^{\alpha }Y\left[\frac{{\partial }^{2}}{\partial {x}^{2}}u+2u-3{u}^{2}\right]\right].$$

This implies that.

26 $$u\left(x,t\right)=\lambda +{Y}^{-1}\left[{v}^{\alpha }Y\left[\frac{{\partial }^{2}}{\partial {x}^{2}}u+2u-3{u}^{2}\right]\right].$$

**Step 3:** ADM defines the solution by an infinite series of linear terms as

27 $$u\left(x,t\right)=\sum_{n=0}^{\infty }{u}_{n}\left(x,t\right),$$

and the nonlinear term as

28 $${u}^{2}=\sum_{n=0}^{\infty }{A}_{n}\left(u\right),$$

where ${A}_{n}\left(u\right)$ are the Adomian polynomials to be determined.

**Step 4:** substituting (28) and (27) into (26) by using the linearity property of the YT, to obtain

29 $$\sum_{n=0}^{\infty }{u}_{n}\left(x,t\right)=\lambda +{Y}^{-1}\left[{v}^{\alpha }Y\left[\sum_{n=0}^{\infty }\frac{{\partial }^{2}}{\partial {x}^{2}}{u}_{n}+2\sum_{n=0}^{\infty }{u}_{n}-3\sum_{n=0}^{\infty }{A}_{n}\left(u\right)\right]\right].$$

**Step 5:** Equating the terms on both sides of (29), we have the following recurrence relation.

$${u}_{0}\left(x,t\right)=\lambda ,$$

30 $${u}_{n+1}\left(x,t\right)={Y}^{-1}\left[{v}^{\alpha }Y\left[\frac{{\partial }^{2}}{\partial {x}^{2}}{u}_{n}\left(x,t\right)+2{u}_{n}\left(x,t\right)-3{A}_{n}\left(u\right)\right]\right], n\ge 0.$$

By using (9), the few components of the Adomian polynomials for the nonlinear term ${u}^{2}$ have been derived in the form

$$A_{0} \left( u \right) = u_{0}^{2} ,\,A_{1} \left( u \right) = 2u_{0} u_{1} ,\,A_{2} \left( u \right) = 2u_{0} u_{2} + u_{1}^{2} ,$$

$$A_{3} \left( u \right) = 2u_{0} u_{3} + 2u_{1} u_{2} ,{ }A_{4} \left( u \right) = 2u_{0} u_{4} + 2u_{1} u_{3} + u_{2}^{2} ,\, {\text{and so on}}.$$

Consequently, solving the above equations, the first few components of the YTADM solution for (30) are derived as follows:

$${u}_{0}\left(x,t\right)=\lambda ,$$

$${u}_{1}\left(x,t\right)={Y}^{-1}\left[{v}^{\alpha }Y\left[\frac{{\partial }^{2}}{\partial {x}^{2}}{u}_{0}+2{u}_{0}-3{A}_{0}\left(u\right)\right]\right],$$

$$={Y}^{-1}\left[{v}^{\alpha }Y\left[2\lambda -3{\lambda }^{2}\right]\right]={Y}^{-1}\left[{2\lambda v}^{\alpha +1}-{3{\lambda }^{2}v}^{\alpha +1}\right]=\frac{{2\lambda t}^{\alpha }}{\Gamma \left(\alpha +1\right)}-\frac{{3{\lambda }^{2}t}^{\alpha }}{\Gamma \left(\alpha +1\right)}.$$

Using the same technique,

$${u}_{2}\left(x,t\right)=\frac{{t}^{2\alpha }}{\Gamma \left(2\alpha +1\right)}\left(4\lambda -18{\lambda }^{2}+18{\lambda }^{3}\right).$$

$${u}_{3}\left(x,t\right)=\frac{{8\lambda t}^{3\alpha }}{\Gamma \left(3\alpha +1\right)}\left(8\lambda -60{\lambda }^{2}+144{\lambda }^{3}-108{\lambda }^{4}-\frac{12{\lambda }^{2}\Gamma \left(2\alpha +1\right)}{{\left(\Gamma \left(\alpha +1\right)\right)}^{2}}+\frac{36{\lambda }^{3}\Gamma \left(2\alpha +1\right)}{{\left(\Gamma \left(\alpha +1\right)\right)}^{2}}-\frac{27{\lambda }^{4}\Gamma \left(2\alpha +1\right)}{{\left(\Gamma \left(\alpha +1\right)\right)}^{2}}\right).$$

$${u}_{4}\left(x,t\right)=\frac{{t}^{4\alpha }}{\Gamma \left(4\alpha +1\right)}\left(16\lambda -168{\lambda }^{2}+648{\lambda }^{3}-1080{\lambda }^{4}+648{\lambda }^{5}-\frac{24{\lambda }^{2}\Gamma \left(2\alpha +1\right)}{{\left(\Gamma \left(\alpha +1\right)\right)}^{2}}+\frac{144{\lambda }^{3}\Gamma \left(2\alpha +1\right)}{{\left(\Gamma \left(\alpha +1\right)\right)}^{2}}- \frac{270{\lambda }^{4}\Gamma \left(2\alpha +1\right)}{{\left(\Gamma \left(\alpha +1\right)\right)}^{2}}+ \frac{144{\lambda }^{3}\Gamma \left(2\alpha +1\right)}{{\left(\Gamma \left(\alpha +1\right)\right)}^{2}}-\frac{270{\lambda }^{4}\Gamma \left(2\alpha +1\right)}{{\left(\Gamma \left(\alpha +1\right)\right)}^{2}}+\frac{162{\lambda }^{5}\Gamma \left(2\alpha +1\right)}{{\left(\Gamma \left(\alpha +1\right)\right)}^{2}}-\frac{48{\lambda }^{2}\Gamma \left(3\alpha +1\right)}{\Gamma \left(\alpha +1\right)\Gamma \left(2\alpha +1\right)}+ \frac{288{\lambda }^{3}\Gamma \left(3\alpha +1\right)}{\Gamma \left(\alpha +1\right)\Gamma \left(2\alpha +1\right)}-\frac{540{\lambda }^{4}\Gamma \left(3\alpha +1\right)}{\Gamma \left(\alpha +1\right)\Gamma \left(2\alpha +1\right)}+\frac{324{\lambda }^{5}\Gamma \left(3\alpha +1\right)}{\Gamma \left(\alpha +1\right)\Gamma \left(2\alpha +1\right)}\right),$$

and so on.

**Step 6:** The numerical solution of YTADM is given as

31 $$\begin{gathered} u\left( {x,t} \right) = u_{0} \left( {x,t} \right) + u_{1} \left( {x,t} \right) + u_{2} \left( {x,t} \right) + \ldots , \hfill \\ = \lambda + \frac{{t^{\alpha } }}{{{\Gamma }\left( {\alpha + 1} \right)}}\left( {2\lambda - 3\lambda^{2} } \right) + \frac{{t^{2\alpha } }}{{{\Gamma }\left( {2\alpha + 1} \right)}}\left( {4\lambda - 18\lambda^{2} + 18\lambda^{3} } \right) + \frac{{t^{3\alpha } }}{{{\Gamma }\left( {3\alpha + 1} \right)}}\left( {8\lambda - 60\lambda^{2} + 144\lambda^{3} - 108\lambda^{4} } \right) + \cdots . \hfill \\ \end{gathered}$$

**Fig. 1:**
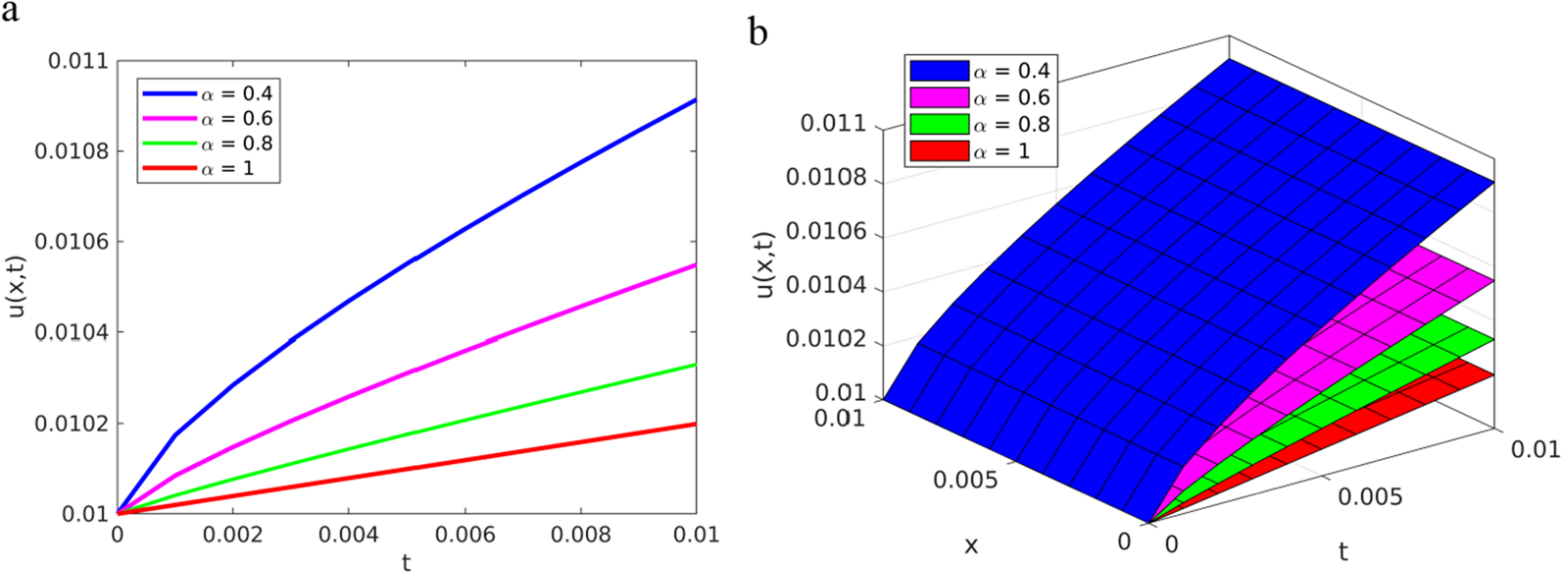
Solution plots of YTADM to Example 4.1 with $\alpha =0.4, 0.6, 0.8, 1$ **a** line plots, **b** surface plots

**Fig. 2:**
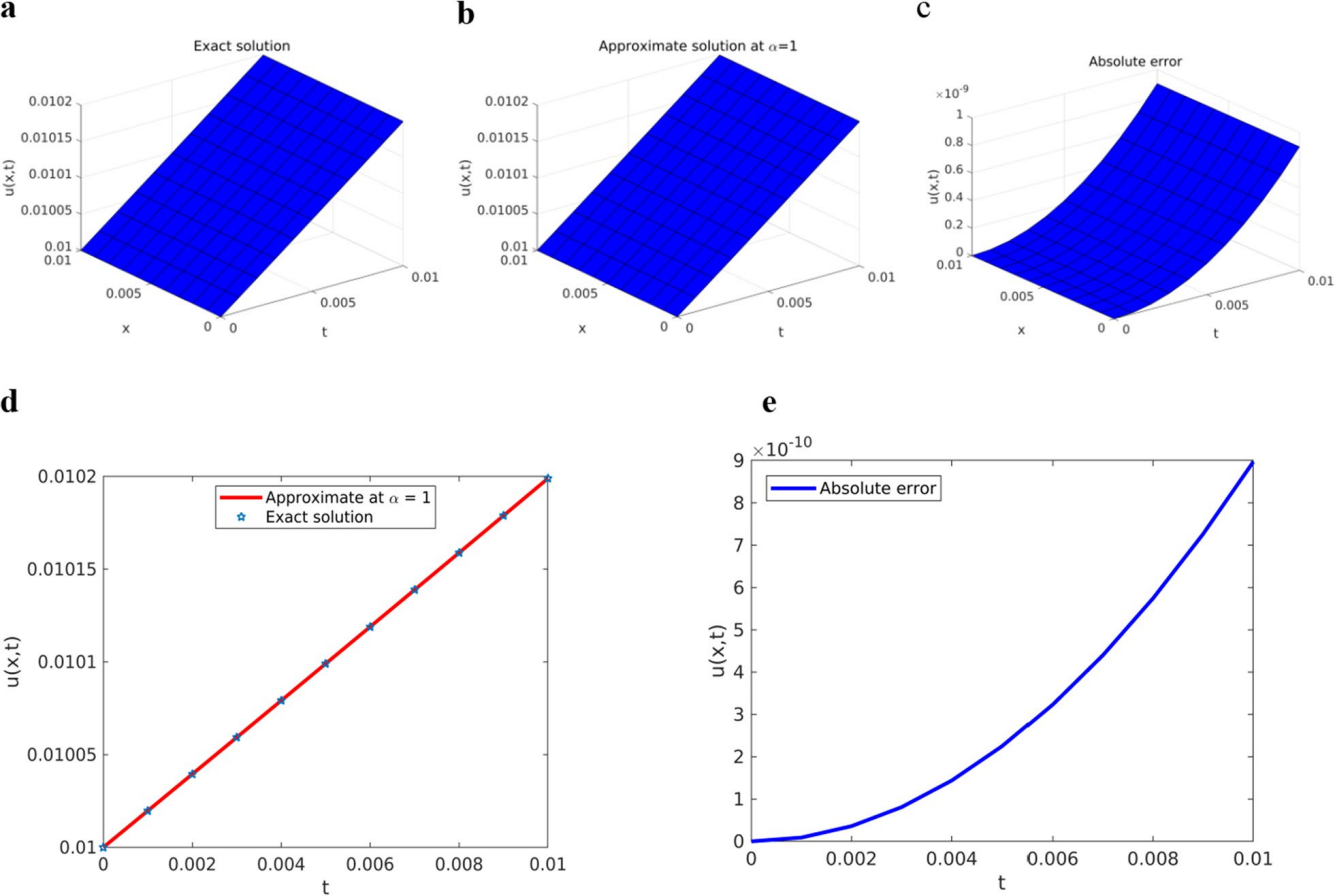
Solution plots of YTADM for Example 4.1**a** surface of the exact solution, **b** surface of the approximate solution, and **c** surface of the absolute error, **d** comparison at $x=1$, **e** absolute error at $x=1$

To verify the solution approximated by the YTADM graphically, we depict the results in Fig. 1 for different values of the fractional order ($\alpha =0.4, 0.6, \text{0.8,1}$) and in Fig. 2 for $\alpha =1$.

As shown in Figs. 1a and b, the approximate solutions of the pattern behavior gradually decrease as the fractional order values get closer and closer to. The same as in the first example, for comparison purposes, we consider only the special case because the exact solution exists. For this reason, in Fig. 2, we compare the approximate solution with the exact solution.

As seen from Fig. 2, the approximate solution obtained is similar to the exact solution. This finding implies that the results obtained by YTADM are in good agreement with the exact results. The numerical solution to this problem is also compared with the results of the Laplace transform decomposition method (LTDM) [64], the Aboodh transform homotopy perturbation method (ATHPM) [65], and the q-homotopy Shehu analysis transform method (q-HSATM) [61] in Table 1 at the same number of iterations and domain discretization.

**Table 1:** Approximate (Approx.) and comparison solutions of YTADM with the exact solution for Example 4.1 at $\lambda =0.01$

$t$	Exact solution	Approx. at $\alpha =0.9$	Approx. at $\alpha =1$	Absolute error at $\alpha =1$		
				YTADM	LTDM [64]	q-HSATM [61] and ATHPM [65]
$0.001$	$0.01001$	$0.01004$	$0.01001$	$9.0021\times {10}^{-12}$	$7.56\times {10}^{-7}$	$1\times {10}^{-12}$
$0.002$	$0.01003$	$0.01007$	$0.01003$	$3.6058\times {10}^{-11}$	$1.51\times {10}^{-6}$	$9\times {10}^{-11}$
$0.003$	$0.01005$	$0.01011$	$0.01005$	$8.1340\times {10}^{-11}$	$2.26\times {10}^{-6}$	$3.2\times {10}^{-10}$
$0.004$	$0.01007$	$0.01014$	$0.01007$	$1.4514\times {10}^{-10}$	$3.02\times {10}^{-6}$	$7.7\times {10}^{-10}$
$0.005$	$0.01009$	$0.01017$	$0.01009$	$2.2788\times {10}^{-10}$	$3.78\times {10}^{-6}$	$1.5\times {10}^{-9}$
$0.006$	$0.01011$	$0.01020$	$0.01011$	$3.3012\times {10}^{-10}$	$4.53\times {10}^{-6}$	$2.6\times {10}^{-9}$
$0.007$	$0.01013$	$0.01023$	$0.01013$	$4.5253\times {10}^{-10}$	$5.20\times {10}^{-6}$	$4.1\times {10}^{-9}$
$0.008$	$0.01015$	$0.01026$	$0.01015$	$5.9590\times {10}^{-10}$	$6.00\times {10}^{-6}$	$6.1\times {10}^{-9}$
$0.009$	$0.01017$	$0.01030$	$0.01017$	$7.6118\times {10}^{-10}$	$6.80\times {10}^{-6}$	$8.7\times {10}^{-9}$
$0.01$	$0.01019$	$0.01033$	$0.01019$	$9.4942\times {10}^{-10}$	$7.05\times {10}^{-6}$	$1.2\times {10}^{-8}$

Table 1 shows the numerical approximate and comparison solutions of YTADM and other numerical methods. The absolute errors of YTADM at various points in the corresponding domain are presented and compared with LTDM [64], q-HSATM [61], ATHPM [65], and the exact solution at $\alpha =1$, it yields far more robust results than all the other numerical methods. Hence, we conclude that the absolute error determined in the table shows that the method is too accurate for treating NTFPDEs.

### ***Example 4.2***

[63] Consider the nonlinear fractional Cauchy reaction–diffusion equation.

32 $${}_{0}{}^{C}{D}_{t}^{\alpha }u\left(x,t\right)=\, {u}_{xx}\left(x,t\right)-{u}_{x}\left(x,t\right)+u\left(x,t\right){u}_{x}\left(x,t\right)-{u}^{2}\left(x,t\right)+u\left(x,t\right), 0<\alpha \le 1$$

with initial condition

$$u\left(x,0\right)={e}^{x}.$$

The exact solution of this problem is $u(x,t)={e}^{x+t}$ for the special case $\alpha =1$. For simplicity we drop $\left(x,t\right)$ from each term of (32). To solve this problem by YTADM, we follow the procedure stated in Sect. "A hybrid numerical method for solving NTFPDEs".

**Step 1:** Apply YT on both sides of (32) to obtain

33 $$\frac{T\left(x,v\right)}{{v}^{\alpha }}-\sum_{k=0}^{m-1}{v}^{k-\alpha +1}{u}^{\left(k\right)}\left(x,0\right)=Y\left[{u}_{xx}-{u}_{x}+u{u}_{x}-{u}^{2}+u\right].$$

In our case $0<\alpha \le 1$, (33) is reduced to

$$\frac{T\left(x,v\right)}{{v}^{\alpha }}={v}^{1-\alpha }u\left(x,0\right)+Y\left[{u}_{xx}-{u}_{x}+u{u}_{x}-{u}^{2}+u\right],$$

34 $$T\left(x,v\right)=v{e}^{x}+{v}^{\alpha }Y\left[{u}_{xx}-{u}_{x}+u{u}_{x}-{u}^{2}+u\right].$$

**Step 2:** Taking the inverse YT on both sides of (34),

35 $$u\left(x,t\right)={e}^{x}+{Y}^{-1}\left[{v}^{\alpha }Y\left[{u}_{xx}-{u}_{x}+u{u}_{x}-{u}^{2}+u\right]\right].$$

**Step 3:** The ADM defines the solution by an infinite series of linear terms as

36 $$u\left(x,t\right)=\sum_{n=0}^{\infty }{u}_{n}\left(x,t\right),$$

and the nonlinear term as

37 $$u\left(x,t\right){u}_{x}\left(x,t\right)=\sum_{n=0}^{\infty }{A}_{n}\left(u\right),$$

38 $${u}^{2}\left(x,t\right)=\sum_{n=0}^{\infty }{B}_{n}\left(u\right),$$

where ${A}_{n}$ and ${B}_{n}$ are the Adomian polynomials to be determined.

**Step 4:** Substituting (36), (37) and (38) into (35) by using the linearity property of the YT, to obtain

39 $$\begin{gathered} \mathop \sum \limits_{n = 0}^{\infty } u_{n} \left( {x,t} \right) = e^{x} + Y^{ - 1} \left[ {v^{\alpha } Y\left[ {\mathop \sum \limits_{n = 0}^{\infty } u_{nxx} } \right]} \right] - Y^{ - 1} \left[ {v^{\alpha } Y\left[ {\mathop \sum \limits_{n = 0}^{\infty } u_{nx} } \right]} \right] + Y^{ - 1} \left[ {v^{\alpha } Y\left[ {\mathop \sum \limits_{n = 0}^{\infty } u_{n} } \right]} \right] \hfill \\ + Y^{ - 1} \left[ {v^{\alpha } Y\left[ {\mathop \sum \limits_{n = 0}^{\infty } A_{n} \left( u \right)} \right]} \right] - Y^{ - 1} \left[ {v^{\alpha } Y\left[ {\mathop \sum \limits_{n = 0}^{\infty } B_{n} \left( u \right)} \right]} \right] \hfill \\ \end{gathered}$$

**Step 5:** To obtain the component solutions, we equating the terms on both sides of (39) to get

$$\begin{aligned} u_{0} \left( {x,t} \right) =\, & e^{x} ,u_{{n + 1}} \left( {x,t} \right) = Y^{{ - 1}} \left[ {v^{\alpha } Y\left[ {u_{{nxx}} } \right]} \right] - Y^{{ - 1}} \left[ {v^{\alpha } Y\left[ {u_{{nx}} } \right]} \right] + Y^{{ - 1}} \left[ {v^{\alpha } Y\left[ {u_{n} } \right]} \right] \\ + & Y^{{ - 1}} \left[ {v^{\alpha } Y\left[ {A_{n} \left( u \right)} \right]} \right] - Y^{{ - 1}} \left[ {v^{\alpha } Y\left[ {B_{n} \left( u \right)} \right]} \right],n \ge 0. \\ \end{aligned}$$

By using (9), the few components of the Adomian polynomials for the nonlinear terms $u{u}_{xx}$ and ${u}^{2}$ have been derived in the form

$$A_{0} \left( u \right) = u_{0} u_{0x} ,\,A_{1} \left( u \right) = u_{0} u_{1x} + u_{1} u_{0x} ,\,A_{2} \left( u \right) = u_{0} u_{2x} + u_{1} u_{1x} + u_{2} u_{0x} ,$$

${A}_{3}\left(u\right)={u}_{0}{u}_{3x}+{u}_{1}{u}_{2x}+{u}_{2}{u}_{1x}+{u}_{3}{u}_{0x},$ and so on.

${B}_{0}\left(u\right)={u}_{0}^{2},$ ${B}_{1}\left(u\right)=2{u}_{0}{u}_{1},$ ${B}_{2}\left(u\right)=2{u}_{0}{u}_{2}+{u}_{1}^{2},$ ${B}_{3}\left(u\right)=2{u}_{0}{u}_{3}+2{u}_{1}{u}_{2},$ and so on.

Since ${u}_{0}\left(x,t\right)={e}^{x}$, the first iteration solution is given by

$$\begin{aligned} u_{1} \left( {x,t} \right) =\, & Y^{{ - 1}} \left[ {v^{\alpha } Y\left[ {u_{{0xx}} } \right]} \right] - Y^{{ - 1}} \left[ {v^{\alpha } Y\left[ {u_{{0x}} } \right]} \right] + Y^{{ - 1}} \left[ {v^{\alpha } Y\left[ {u_{0} } \right]} \right] \\ + & Y^{{( - 1)}} [v^{\alpha } Y[A_{0} (u)]] - Y^{{( - 1)}} [v^{\alpha } Y[B_{0} (u)]], \\ = & Y^{{( - 1)}} [v^{\alpha } Y[e^{x} ]] = Y^{{( - 1)}} [v^{\alpha } (ve^{x} )] = e^{x} Y^{{ - 1}} \left[ {v^{{\alpha + 1}} } \right] = \frac{{e^{x} t^{\alpha } }}{{\Gamma \left( {\alpha + 1} \right)}} \\ \end{aligned}$$

Using the same technique, we obtain.

${u}_{2}\left(x,t\right)=\frac{{e}^{x}{t}^{2\alpha }}{\Gamma \left(2\alpha +1\right)}$, ${u}_{3}\left(x,t\right)=\frac{{e}^{x}{t}^{3\alpha }}{\Gamma \left(3\alpha +1\right)}$, ${u}_{4}\left(x,t\right)=\frac{{e}^{x}{t}^{4\alpha }}{\Gamma \left(4\alpha +1\right)}$ and so on.

**Step 6:** Following component approximate solution, we obtain the general solution as

40 $$\begin{aligned} u\left( {x,t} \right) = \,& u_{0} \left( {x,t} \right) + u_{1} \left( {x,t} \right) + u_{2} \left( {x,t} \right) + \cdots , \\ =\, & e^{x} \left( {1 + \frac{{t^{\alpha } }}{{{\Gamma }\left( {\alpha + 1} \right)}} + \frac{{t^{2\alpha } }}{{{\Gamma }\left( {2\alpha + 1} \right)}} + \frac{{t^{3\alpha } }}{{{\Gamma }\left( {3\alpha + 1} \right)}} + \frac{{t^{4\alpha } }}{{{\Gamma }\left( {4\alpha + 1} \right)}} + \ldots } \right) = e^{x} E_{\alpha } \left( {t^{\alpha } } \right), \\ \end{aligned}$$

where ${\text{E}}_{\alpha } \left( . \right)$ is well-known as the Mittag–Leffler function defined in [7]*.*

**Fig. 3:**
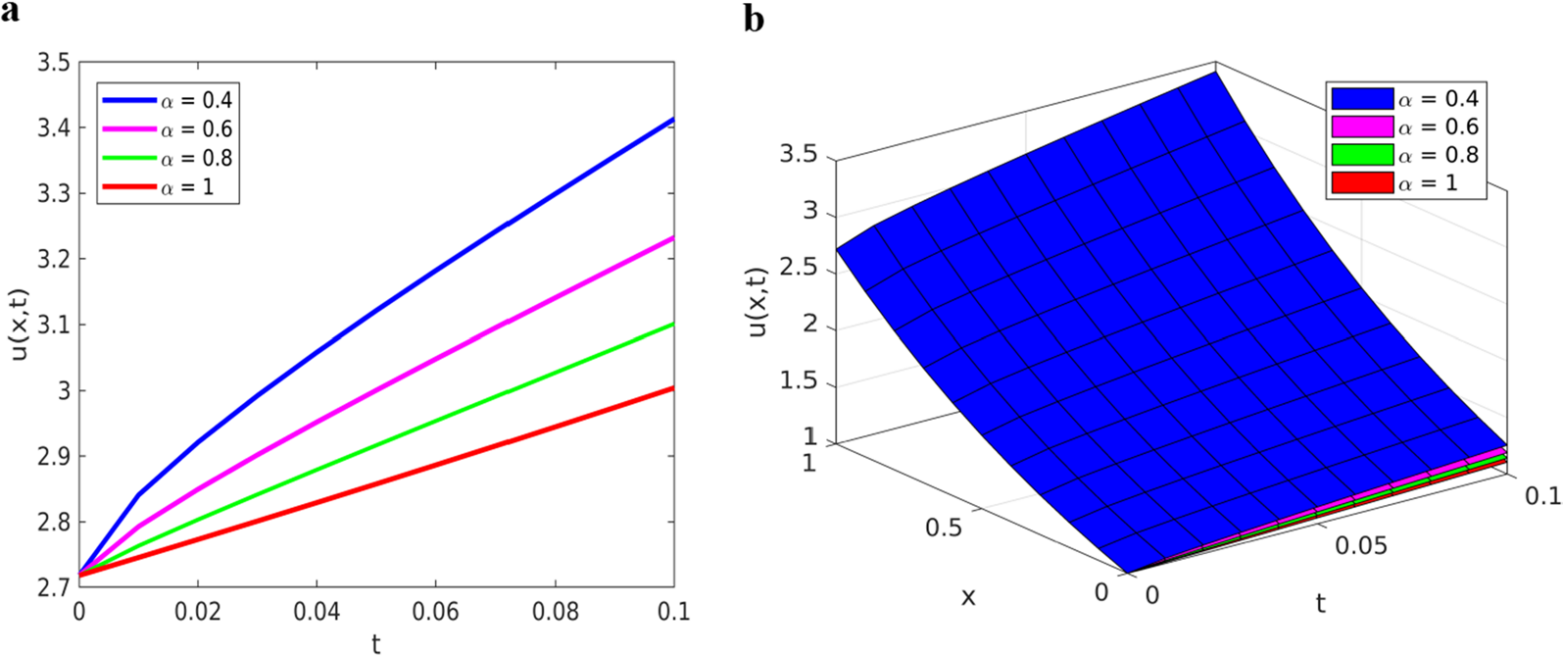
Solution plots of YTADM for Example 4.2 with $\alpha =0.4, 0.6, 0.8, 1$; **a** line plots at $x=1$ for $0\le t\le 1$, **b** surface plots for $0\le x,t\le 1$

**Fig. 4:**
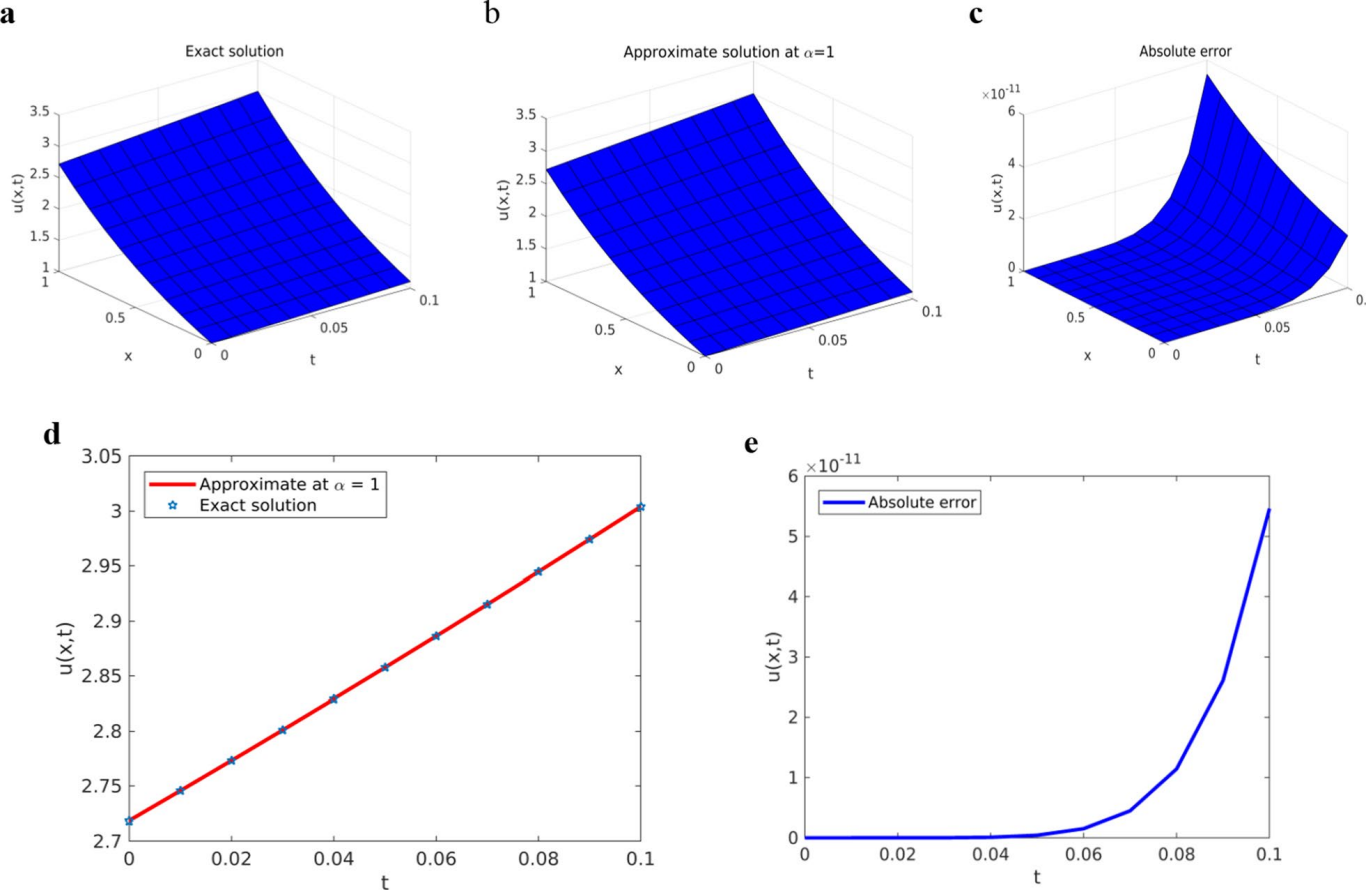
Solution plots of YTADM for Example 4.2**a** surface of the exact solution, **b** surface of the approximate solution, and **c** surface of the absolute error, **d** comparison at $x=1$, **e** absolute error at $x=1$

To show the solution behavior of the approximate solution obtained by YTADM, we consider different values of the fractional order $\alpha$ $\left(\alpha =0.4, 0.6, 0.8, 1\right)$ and depict the results in Figs. 3 and 4.

The numerical solutions obtained by YTADM for distinct fractional orders are depicted in Figs. 3a and b. We have observed from this figure that the solution behaviors of the time-fractional derivatives are clearly decreasing as the values of fractional order increase. In the case of ordinary derivatives, the solution behavior of such a DE is not clearly shown. Therefore, arbitrariness in fractional-order derivatives introduces more degrees of freedom in the design and study of real-time events. It is observed from Figs. 4b and d that the approximate solution obtained by YTADM is almost identical to the exact solution at. From Fig. 4, it is found that exact and approximate solutions are in complete agreement. The absolute errors illustrated in Figs. 4c and e indicate that the designed numerical method is a suitable one for solving nonlinear fractional Cauchy reaction–diffusion equations that arise in various fields of science. We have also compared YTADM with the Aboodh variational iteration method (AVIM) [63] using their absolute errors. For this reason, we consider the time discretization points as in AVIM and record the obtained results in Table 2.

**Table 2:** Approximate (Approx.) and comparison solutions of YTADM, AVIM [63] and the exact solution for Example 4.2 when $x=1$

$t$	Exact solution	Approx. at $\alpha =0.8$	Approx. at $\alpha =0.9$	Approx. at $\alpha =1$	Absolute error at $\alpha =1$	
					YTADM	AVIM [63]
$0.01$	$2.745601$	$2.792806$	$2.763486$	$2.745601$	$8.8817{\times 10}^{-16}$	$2.2690{\times 10}^{-12}$
$0.03$	$2.801065$	$2.901994$	$2.841677$	$2.801065$	$1.1990{\times 10}^{-14}$	$5.5322{\times 10}^{-10}$
$0.05$	$2.857651$	$3.000419$	$2.916542$	$2.857651$	$4.2410{\times 10}^{-13}$	$7.1383{\times 10}^{-9}$
$0.07$	$2.915379$	$3.094620$	$2.990430$	$2.915379$	$4.4813{\times 10}^{-12}$	$3.8520{\times 10}^{-8}$
$0.09$	$2.974274$	$3.186785$	$3.064180$	$2.974274$	$2.6088{\times 10}^{-11}$	$1.3579{\times 10}^{-7}$

Table 2 shows the numerical solution of Example 4.2 for different points in the domain with different values of fractional order. For the special case of fractional order $\alpha =1$, we compared the numerical solution obtained by YTADM with the solution in the [63] and the exact solution. The obtained result indicates that the solution obtained by YTADM has a better agreement with the exact solution than the approximate solution obtained by AVIM. One of the drawbacks of an AVIM in [63] is that the author only considered the first four iterations. It is well known that to get accurate solution considering more number of iterations is advisable. For this example, we considered the first six terms of the solution components and we obtained a more accurate solution than the one found in [63] as shown in Table 2.

### ***Example 4.3***

[50] Consider the following nonlinear time-fractional Fornberg-Whitham equation:

41 $${}_{0}^{C} D_{t}^{\alpha } u\left( {x,t} \right) =\, u_{xxt} \left( {x,t} \right) - u_{x} \left( {x,t} \right) + u\left( {x,t} \right)u_{xxx} \left( {x,t} \right) - u\left( {x,t} \right)u_{x} \left( {x,t} \right) + 3u_{x} \left( {x,t} \right)u_{xx} \left( {x,t} \right),\quad 0 < \alpha \le 1,$$

with initial conditions

$$u\left(x,0\right)={e}^\frac{x}{2}.$$

The exact solution of this problem is $u(x,t)={e}^{\frac{x}{2}-\frac{2t}{3}}$ for the special case $\alpha =1$.

**Step 1:** Applying YT on both sides of (41) and using the differentiation property, we have.

42 $$\frac{T\left(x,v\right)}{{v}^{\alpha }}-\sum_{k=0}^{m-1}{v}^{k-\alpha +1}{u}^{\left(k\right)}\left(x,0\right)=Y\left[{u}_{xxt}-{u}_{x}+u{u}_{xxx}-u{u}_{x}+3{u}_{x}{u}_{xx}\right].$$

In our case $0<\alpha \le 1,$ (42) is reduced to

43 $$\frac{T\left(x,v\right)}{{v}^{\alpha }}=vu\left(x,0\right)+Y\left[{u}_{xxt}-{u}_{x}+u{u}_{xxx}-u{u}_{x}+3{u}_{x}{u}_{xx}\right].$$

**Step 2:** Applying the inverse YT on both sides of (43), we obtain.

44 $$u\left(x,t\right)={e}^\frac{x}{2}+{Y}^{-1}\left[{v}^{\alpha }Y\left[{u}_{xxt}-{u}_{x}+u{u}_{xxx}-u{u}_{x}+3{u}_{x}{u}_{xx}\right]\right].$$

**Step 3:** ADM defines the solution by an infinite series of linear terms as

45 $$u\left(x,t\right)=\sum_{n=0}^{\infty }{u}_{n}\left(x,t\right),$$

and the nonlinear term as

46 $$u\left(x,t\right){u}_{xxx}\left(x,t\right)=\sum_{n=0}^{\infty }{A}_{n}\left(u\right),$$

47 $$u\left(x,t\right){u}_{x}\left(x,t\right)=\sum_{n=0}^{\infty }{B}_{n}\left(u\right),$$

48 $${u}_{x}\left(x,t\right){u}_{xx}\left(x,t\right)=\sum_{n=0}^{\infty }{C}_{n}\left(u\right),$$

where ${A}_{n}, {B}_{n}$ and ${C}_{n}$ are the Adomian polynomials to be determined.

**Step 4:** Using the linearity property of the YT and substituting (45)-(48) into (44), we obtain

49 $$\begin{aligned} \mathop \sum \limits_{n = 0}^{\infty } u_{n} \left( {x,t} \right) =\, & e^{\frac{x}{2}} + Y^{ - 1} \left[ {v^{\alpha } Y\left[ {\mathop \sum \limits_{n = 0}^{\infty } u_{nxxt} } \right]} \right] - Y^{ - 1} \left[ {v^{\alpha } Y\left[ {\mathop \sum \limits_{n = 0}^{\infty } u_{nx} } \right]} \right] + Y^{ - 1} \left[ {v^{\alpha } Y\left[ {\mathop \sum \limits_{n = 0}^{\infty } A_{n} \left( u \right)} \right]} \right] \\ - & Y^{ - 1} \left[ {v^{\alpha } Y\left[ {\mathop \sum \limits_{n = 0}^{\infty } B_{n} \left( u \right)} \right]} \right] + 3Y^{ - 1} \left[ {v^{\alpha } Y\left[ {\mathop \sum \limits_{n = 0}^{\infty } C_{n} \left( u \right)} \right]} \right] \\ \end{aligned}$$

**Step 5:** Equating the terms on both sides of (49), we have the following relation

$$\begin{gathered} u_{0} \left( {x,t} \right) = e^{\frac{x}{2}} , \hfill \\ u_{n + 1} \left( {x,t} \right) = Y^{ - 1} \left[ {v^{\alpha } Y\left[ {u_{nxxt} } \right]} \right] - Y^{ - 1} \left[ {v^{\alpha } Y\left[ {u_{nx} } \right]} \right] + Y^{ - 1} \left[ {v^{\alpha } Y\left[ {A_{n} \left( u \right)} \right]} \right] \hfill \\ - Y^{ - 1} \left[ {v^{\alpha } Y\left[ {B_{n} \left( u \right)} \right]} \right] + 3Y^{ - 1} \left[ {v^{\alpha } Y\left[ {C_{n} \left( u \right)} \right]} \right], n \ge 0. \hfill \\ \end{gathered}$$

By using (9), the few components of the Adomian polynomials for the nonlinear terms $u{u}_{xxx}, u{u}_{x}$ and ${u}_{x}{u}_{xx}$ have been derived in the form

$$A_{0} \left( u \right) = u_{0} u_{0xxx} ,\,A_{1} \left( u \right) = u_{0} u_{1xxx} + u_{1} u_{0xxx} ,$$

$${A}_{2}\left(u\right)={u}_{0}{u}_{2xxx}+{u}_{1}{u}_{1xxx}+{u}_{2}{u}_{0xxx},$$

${A}_{3}\left(u\right)={u}_{0}{u}_{3xxx}+{u}_{1}{u}_{2xxx}+{u}_{2}{u}_{1xxx}+{u}_{3}{u}_{0xxx},$ and so on.

$$B_{0} \left( u \right) = u_{0} u_{0x} ,\,B_{1} \left( u \right) = u_{0} u_{1x} + u_{1} u_{0x} ,\,B_{2} \left( u \right) = u_{0} u_{2x} + u_{1} u_{1x} + u_{2} u_{0x} ,$$

${B}_{3}\left(u\right)={u}_{0}{u}_{3x}+{u}_{1}{u}_{2x}+{u}_{2}{u}_{1x}+{u}_{3}{u}_{0x},$ and so on.

$$C_{0} \left( u \right) = u_{0x} u_{0xx} ,\,C_{1} \left( u \right) = u_{0x} u_{1xx} + u_{1x} u_{0xx} ,\,C_{2} \left( u \right) = u_{0x} u_{2xx} + u_{1x} u_{1xx} + u_{2x} u_{0xx} ,$$

${C}_{3}\left(u\right)={u}_{0x}{u}_{3xx}+{u}_{1x}{u}_{2xx}+{u}_{2x}{u}_{1xx}+{u}_{3x}{u}_{0xx},$ and so on.

Then the iteration solution of YTADM is obtained as follows

$${u}_{0}\left(x,t\right)={e}^\frac{x}{2},$$

$$\begin{aligned} u_{1} \left( {x,t} \right) = & Y^{{ - 1}} \left[ {v^{\alpha } Y\left[ {u_{{0xxt}} } \right]} \right] - Y^{{ - 1}} \left[ {v^{\alpha } Y\left[ {u_{{0x}} } \right]} \right] + Y^{{ - 1}} \left[ {v^{\alpha } Y\left[ {A_{0} \left( u \right)} \right]} \right] - Y^{{ - 1}} \left[ {v^{\alpha } Y\left[ {B_{0} \left( u \right)} \right]} \right] + 3Y^{{ - 1}} \left[ {v^{\alpha } Y\left[ {C_{0} \left( u \right)} \right]} \right], \\ = & - Y^{{ - 1}} \left[ {v^{\alpha } Y\left[ {\frac{1}{2}e^{{\frac{x}{2}}} } \right]} \right] + Y^{{ - 1}} \left[ {v^{\alpha } Y\left[ {\frac{1}{8}e^{x} } \right]} \right] - Y^{{ - 1}} \left[ {v^{\alpha } Y\left[ {\frac{1}{2}e^{x} } \right]} \right] + 3Y^{{ - 1}} \left[ {v^{\alpha } Y\left[ {\frac{1}{8}e^{x} } \right]} \right], = - Y^{{ - 1}} \left[ {v^{\alpha } Y\left[ {\frac{1}{2}e^{{\frac{x}{2}}} } \right]} \right] \\ = & - Y^{{ - 1}} \left[ {v^{\alpha } \left( {\frac{v}{2}e^{{\frac{x}{2}}} } \right)} \right] = - Y^{{ - 1}} \left[ {\frac{{v^{{\alpha + 1}} }}{2}e^{{\frac{x}{2}}} } \right] = - \frac{1}{2}e^{{\frac{x}{2}}} \frac{{t^{\alpha } }}{{\Gamma \left( {\alpha + 1} \right)}} \\ \end{aligned}$$

In a similar manner, we obtain.

$${u}_{2}\left(x,t\right)=-\frac{1}{8}{e}^\frac{x}{2}\frac{{t}^{2\alpha -1}}{\Gamma \left(2\alpha \right)}+\frac{1}{4}{e}^\frac{x}{2}\frac{{t}^{2\alpha }}{\Gamma \left(2\alpha +1\right)}.$$

$${u}_{3}\left(x,t\right)=-\frac{1}{32}{e}^\frac{x}{2}\frac{{t}^{3\alpha -2}}{\Gamma \left(3\alpha -1\right)}+\frac{1}{8}{e}^\frac{x}{2}\frac{{t}^{3\alpha -1}}{\Gamma \left(3\alpha \right)}-\frac{1}{8}{e}^\frac{x}{2}\frac{{t}^{3\alpha }}{\Gamma \left(3\alpha +1\right)},$$

${u}_{4}\left(x,t\right)=\frac{1}{16}{e}^\frac{x}{2}\frac{{t}^{4\alpha }}{\Gamma \left(4\alpha +1\right)}-\frac{3}{32}{e}^\frac{x}{2}\frac{{t}^{4\alpha -1}}{\Gamma \left(4\alpha \right)}+\frac{3}{64}{e}^\frac{x}{2}\frac{{t}^{4\alpha -2}}{\Gamma \left(4\alpha -1\right)}-\frac{1}{128}{e}^\frac{x}{2}\frac{{t}^{4\alpha -3}}{\Gamma \left(4\alpha -2\right)},$ and so on.

**Step 6:** The numerical solution of YTADM is given as

50 $$\begin{aligned} u\left( {x,t} \right) = & u_{0} \left( {x,t} \right) + u_{1} \left( {x,t} \right) + u_{2} \left( {x,t} \right) + \ldots , \\ = & e^{{\frac{x}{2}}} - \frac{1}{2}e^{{\frac{x}{2}}} \frac{{t^{\alpha } }}{{\Gamma \left( {\alpha + 1} \right)}} - \frac{1}{8}e^{{\frac{x}{2}}} \frac{{t^{{2\alpha - 1}} }}{{\Gamma \left( {2\alpha } \right)}} + \frac{1}{4}e^{{\frac{x}{2}}} \frac{{t^{{2\alpha }} }}{{\Gamma \left( {2\alpha + 1} \right)}} - \frac{1}{{32}}e^{{\frac{x}{2}}} \frac{{t^{{3\alpha - 2}} }}{{\Gamma \left( {3\alpha - 1} \right)}} + \frac{1}{8}e^{{\frac{x}{2}}} \frac{{t^{{3\alpha - 1}} }}{{\Gamma \left( {3\alpha } \right)}} - \frac{1}{8}e^{{\frac{x}{2}}} \frac{{t^{{3\alpha }} }}{{\Gamma \left( {3\alpha + 1} \right)}} + \ldots \\ \end{aligned}$$

**Fig. 5:**
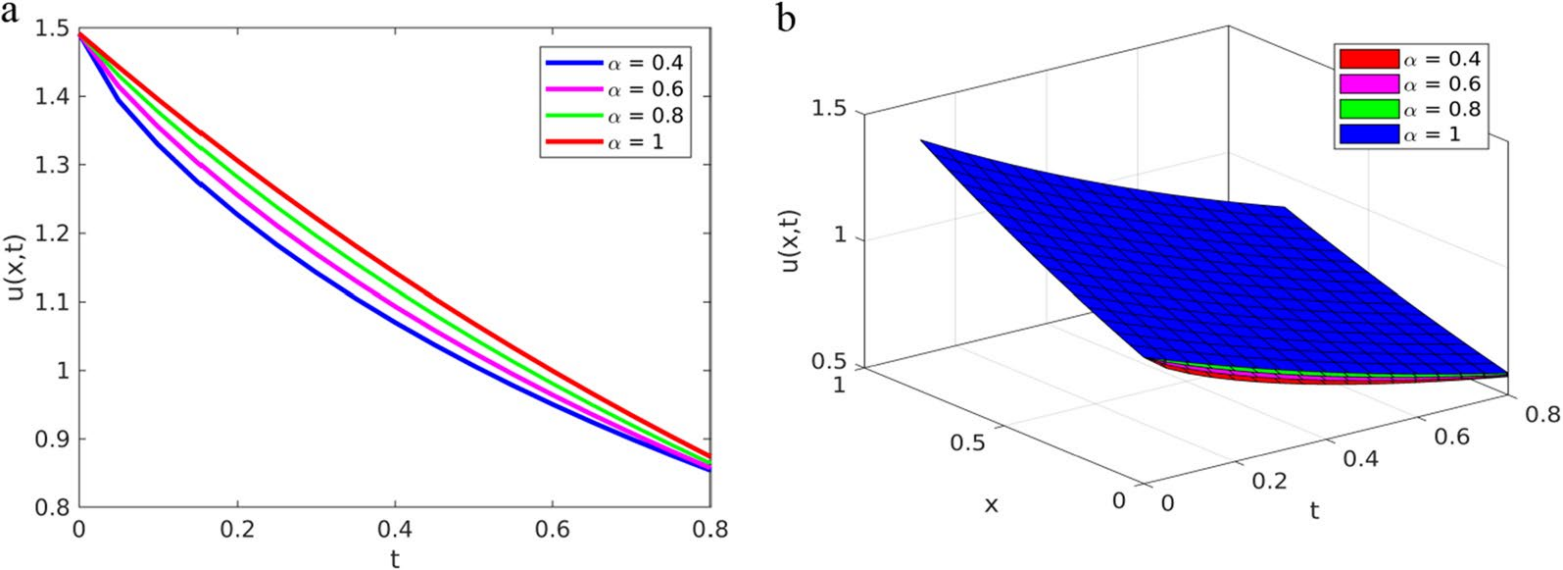
Solution plots of YTADM for Example 4.3 with $\alpha =0.75, 0.85, 0.95, 1$ **a** line plots, **b** surface plots

**Fig. 6:**
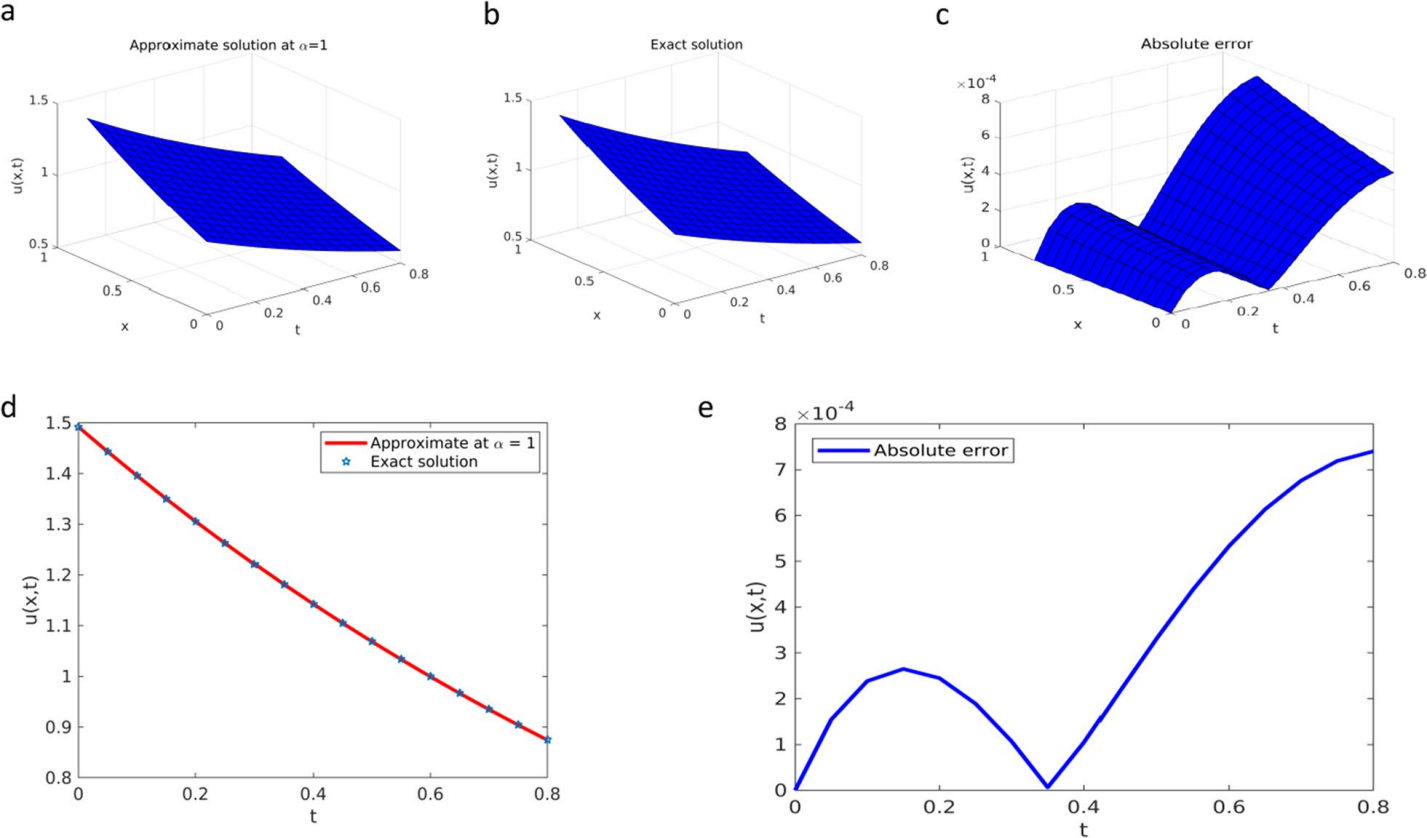
Solution plots of YTADM for Example 4.3**a** surface of the exact solution, **b** surface of the approximate solution, and **c** surface of the absolute error, **d** comparison at $x=1$, **e** absolute error at $x=1$

Here we also investigated the behavior of the approximated solution by up to three iterations of YTADM given in (50) by varying the values of the time-fractional order ($\alpha =0.75, 0.85, 0.95, 1$), and the results are presented in Fig. 5, and the comparison in Fig. 6 at $\alpha =1$

Figure 6 shows the comparison of the numerical solution obtained by YTADM and the exact solution in the 3D and 2D plots of Example 4.3. The 3D and 2D plots have confirmed the closed contact between the YTADM and the exact solutions of Example 4.3.

In Table 3 we compared the obtained results of YTADM with the results of the residual power series method (RPSM) found in [50]. The results show that, with error extremely close to zero, our technique provides a superior answer than the numerical method in [50].

**Table 3:** Approximate and comparison solutions of YTADM, and the exact solution for Example 4.3 when $t=0.01$

$x$	Exact solution	Approx. at $\alpha =0.8$	Approx. at $\alpha =0.9$	Approx. at $\alpha =1$	Absolute error at $\alpha =1$	
					YTADM	RPSM [50]
$-4$	$0.134436$	$0.131160$	$0.133516$	$0.134439$	$3.3733{\times 10}^{-6}$	$2.9899{\times 10}^{-4}$
$-3$	$0.221647$	$0.216247$	$0.220130$	$0.221653$	$5.5617{\times 10}^{-6}$	$4.9296{\times 10}^{-4}$
$-2$	$0.365435$	$0.356532$	$0.362934$	$0.365444$	${9.1697\times 10}^{-6}$	$8.1275{\times 10}^{-4}$
$-1$	$0.602500$	$0.587822$	$0.598378$	$0.602515$	$1.5118{\times 10}^{-5}$	$1.3400{\times 10}^{-3}$
$0$	$0.993355$	$0.969155$	$0.986558$	$0.993380$	$2.4926{\times 10}^{-5}$	$2.2092{\times 10}^{-3}$
$1$	$1.637766$	$1.597867$	$1.626560$	$1.637807$	$4.1096{\times 10}^{-5}$	$3.6425{\times 10}^{-3}$
$2$	$2.700220$	$2.634438$	$2.681744$	$2.700287$	$6.7756{\times 10}^{-5}$	$6.0054{\times 10}^{-3}$
$3$	$4.451910$	$4.343455$	$4.421448$	$4.452022$	$1.1171{\times 10}^{-4}$	$9.9038{\times 10}^{-3}$
$4$	$7.339959$	$7.161147$	$7.289736$	$7.340143$	$1.8418{\times 10}^{-4}$	$1.6324{\times 10}^{-2}$

### ***Example 4.4***

[62] Consider the following nonlinear time-fractional Fokker–Planck equation:

51 $${}_{0}^{C} D_{t}^{\alpha } u\left( {x,t} \right) = - \frac{{\partial \left( {\frac{{4\left( {u\left( {x,t} \right)} \right)^{2} }}{x} - \frac{{xu\left( {x,t} \right)}}{3}} \right)}}{\partial x} + \frac{{\partial^{2} \left( {u\left( {x,t} \right)} \right)^{2} }}{{\partial x^{2} }},\quad 0 < \alpha \le 1,$$

with initial conditions

$$u\left(x,0\right)={x}^{2}.$$

The exact solution of this problem is $u(x,t)={x}^{2}{e}^{t}$ for the special case $\alpha =1$.

**Step 1:** Applying YT on both sides of (51), we obtain.

52 $$\frac{T\left(x,v\right)}{{v}^{\alpha }}-\sum_{k=0}^{m-1}{v}^{k-\alpha +1}{u}^{\left(k\right)}\left(x,0\right)=Y\left[-\frac{\partial \left(\frac{4{\left(u\left(x,t\right)\right)}^{2}}{x}-\frac{xu\left(x,t\right)}{3}\right)}{\partial x}+\frac{{\partial }^{2}{\left(u\left(x,t\right)\right)}^{2}}{\partial {x}^{2}}\right].$$

In our case,$0<\alpha \le 1$, then, (52) is reduced to.

53 $$\frac{T\left(x,v\right)}{{v}^{\alpha }}=vu\left(x,0\right)+Y\left[-\frac{\partial \left(\frac{4{\left(u\left(x,t\right)\right)}^{2}}{x}-\frac{xu\left(x,t\right)}{3}\right)}{\partial x}+\frac{{\partial }^{2}{\left(u\left(x,t\right)\right)}^{2}}{\partial {x}^{2}}\right].$$

**Step 2:** Applying the inverse YT on both sides of (53), to get.

54 $$u\left(x,t\right)={x}^{2}+{Y}^{-1}\left[{v}^{\alpha }Y\left[-\frac{\partial \left(\frac{4{\left(u\left(x,t\right)\right)}^{2}}{x}-\frac{xu\left(x,t\right)}{3}\right)}{\partial x}+\frac{{\partial }^{2}{\left(u\left(x,t\right)\right)}^{2}}{\partial {x}^{2}}\right]\right].$$

**Step 3:** The ADM defines the solution by an infinite series of linear terms as

55 $$u\left(x,t\right)=\sum_{n=0}^{\infty }{u}_{n}\left(x,t\right),$$

and the nonlinear terms as

56 $${\left(u\left(x,t\right)\right)}^{2}=\sum_{n=0}^{\infty }{A}_{n}\left(u\right),$$

where ${A}_{n}$ is the Adomian polynomial to be determined.

**Step 4:** Substituting (55) and (56) into (54), we obtain.

57 $$\sum_{n=0}^{\infty }{u}_{n}\left(x,t\right)={x}^{2}+{Y}^{-1}\left[{v}^{\alpha }Y\left[-\frac{\partial \left(\frac{4}{x}\sum_{n=0}^{\infty }{A}_{n}\left(u\right)-\frac{x}{3}\sum_{n=0}^{\infty }{u}_{n}\right)}{\partial x}+\frac{{\partial }^{2}}{\partial {x}^{2}}\sum_{n=0}^{\infty }{A}_{n}\left(u\right)\right]\right].$$

**Step 5:** Equating the terms on both sides of (57), we have the following relation.

$${u}_{0}\left(x,t\right)={x}^{2},$$

$${u}_{n+1}\left(x,t\right)={Y}^{-1}\left[{v}^{\alpha }Y\left[\frac{\partial \left(\frac{4}{x}\sum_{n=0}^{\infty }{A}_{n}\left(u\right)-\frac{x}{3}\sum_{n=0}^{\infty }{u}_{n}\right)}{\partial x}+\frac{{\partial }^{2}}{\partial {x}^{2}}\sum_{n=0}^{\infty }{A}_{n}\left(u\right)\right]\right], n\ge 0.$$

By using (9), the few components of the Adomian polynomials for the nonlinear term ${u}^{2}$ have been derived as

$$A_{0} \left( u \right) = u_{0}^{2} ,\,A_{1} \left( u \right) = 2u_{0} u_{1} ,\,A_{2} \left( u \right) = 2u_{0} u_{2} + u_{1}^{2} ,$$

$${A}_{3}\left(u\right)={2u}_{0}{u}_{3}+{2u}_{1}{u}_{2},{A}_{4}\left(u\right)=2{u}_{0}{u}_{4}+2{u}_{1}{u}_{3}+{{u}_{2}}^{2}, \text{and so on}.$$

Then, the iteration solution of YTADM is obtained as follows

$${u}_{0}\left(x,t\right)={x}^{2},$$

$$\begin{aligned} u_{1} \left( {x,t} \right) = & Y^{{ - 1}} \left[ {v^{\alpha } Y\left[ { - \frac{{\partial \left( {\frac{4}{x}A_{0} \left( u \right) - \frac{x}{3}u_{0} } \right)}}{{\partial x}} + \frac{{\partial ^{2} }}{{\partial x^{2} }}A_{0} \left( u \right)} \right]} \right], \\ = & Y^{{ - 1}} \left[ {v^{\alpha } Y\left[ { - \frac{{\partial \left( {4x^{3} - \frac{{x^{3} }}{3}} \right)}}{{\partial x}} + \frac{{\partial ^{2} }}{{\partial x^{2} }}\left( {x^{4} } \right)} \right]} \right] = Y^{{ - 1}} \left[ {v^{\alpha } Y\left[ {\frac{{\partial \left( {\frac{{11}}{3}x^{3} } \right)}}{{\partial x}} + 12x^{2} } \right]} \right], \\ = & Y^{{ - 1}} \left[ {v^{\alpha } Y\left[ {x^{2} } \right]} \right] = Y^{{ - 1}} \left[ {v^{\alpha } \left( {vx^{2} } \right)} \right] = Y^{{ - 1}} \left[ {x^{2} v^{{\alpha + 1}} } \right] = x^{2} \frac{{t^{\alpha } }}{{\Gamma \left( {\alpha + 1} \right)}}. \\ \end{aligned}$$

In a similar manner, we obtain.

$${u}_{2}\left(x,t\right)={x}^{2}\frac{{t}^{2\alpha }}{\Gamma \left(2\alpha +1\right)}.$$

${u}_{3}\left(x,t\right)={x}^{2}\frac{{t}^{3\alpha }}{\Gamma \left(3\alpha +1\right)},$ And so on.

**Step 6:** The numerical solution of YTADM is given as

58 $$\begin{gathered} u\left( {x,t} \right) = u_{0} \left( {x,t} \right) + u_{1} \left( {x,t} \right) + u_{2} \left( {x,t} \right) + \cdots , \hfill \\ = x^{2} \left( {1 + \frac{{t^{\alpha } }}{{{\Gamma }\left( {\alpha + 1} \right)}} + \frac{{t^{2\alpha } }}{{{\Gamma }\left( {2\alpha } \right)}} + \frac{{t^{3\alpha } }}{{{\Gamma }\left( {3\alpha + 1} \right)}} + \ldots } \right) \hfill \\ \end{gathered}$$

**Fig. 7:**
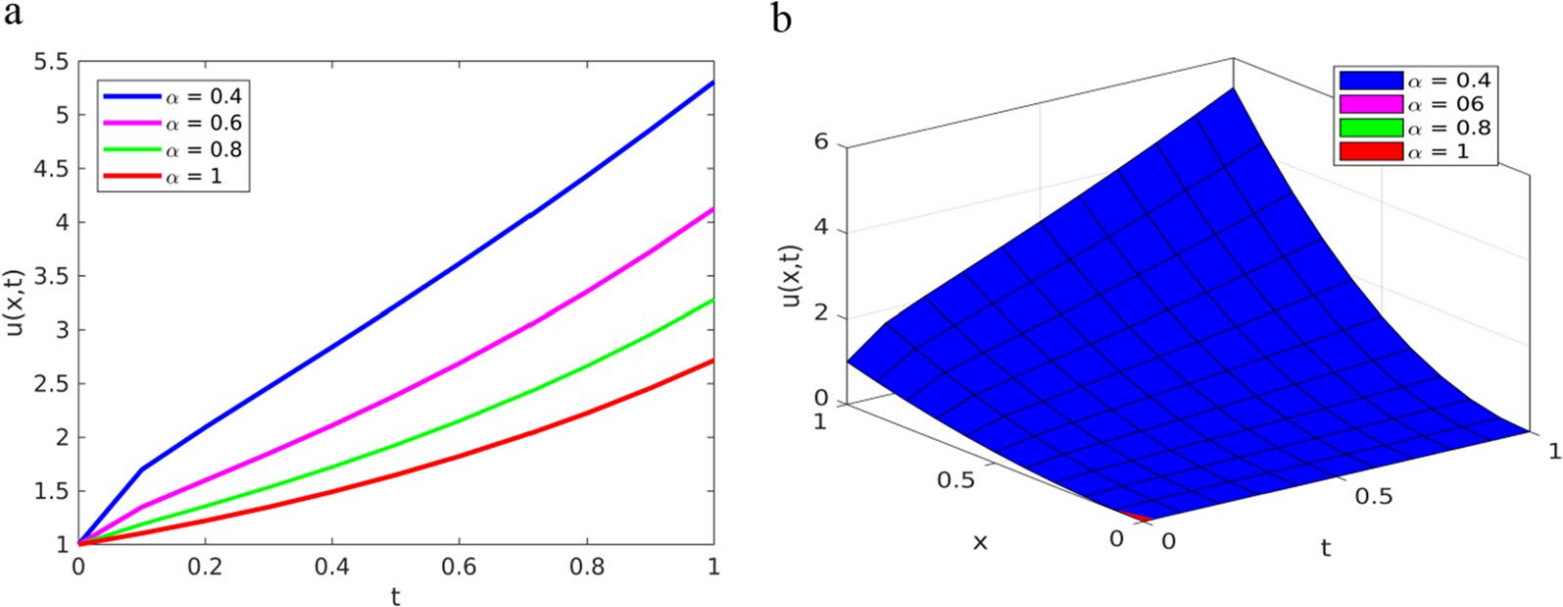
Solution plots of YTADM for Example 4.4 with $\alpha =0.75, 0.85, 0.95, 1, n=4$; **a** line plots, **b** surface plots

**Fig. 8:**
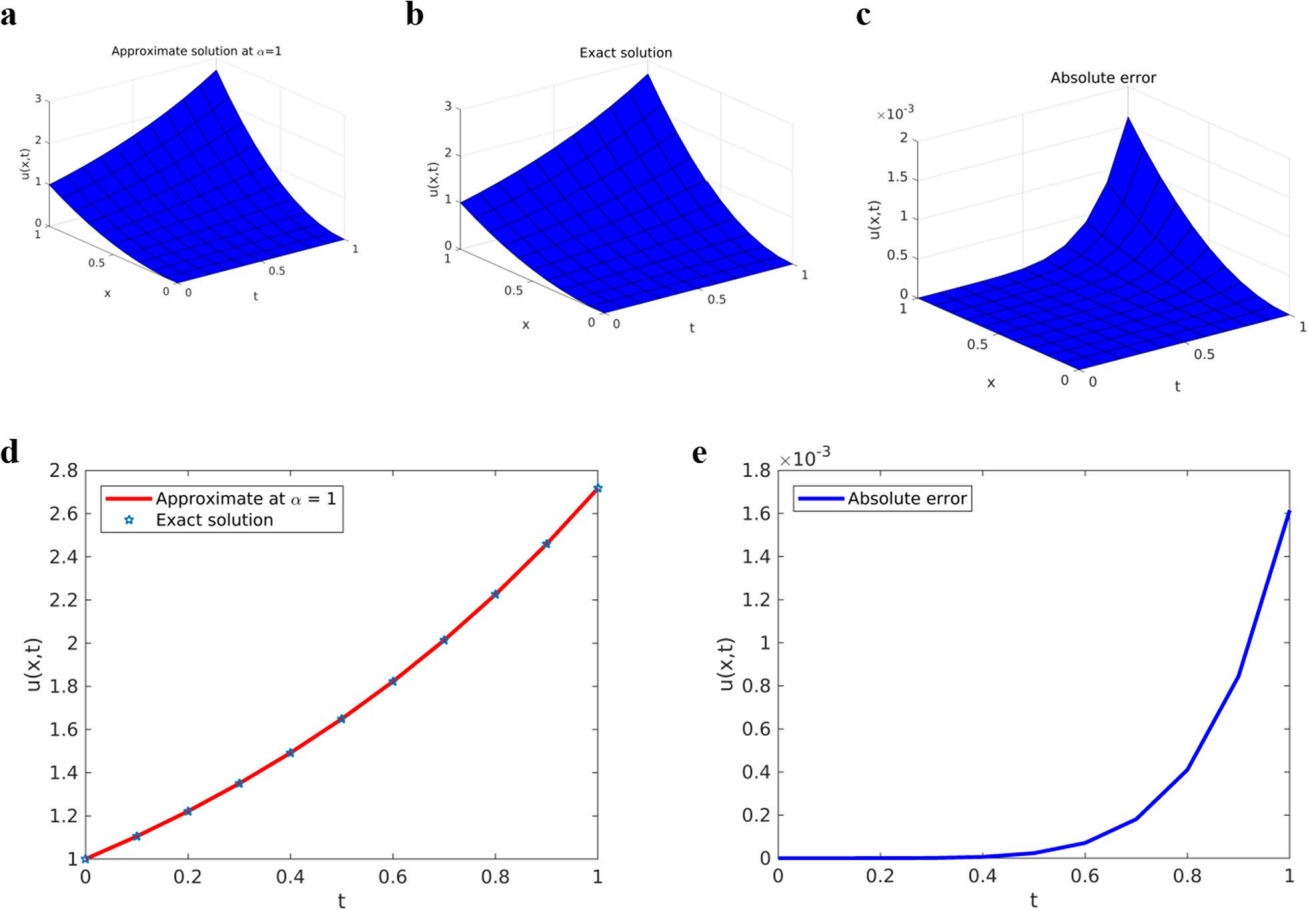
Solution plots of YTADM for Example 4.4**a**) surface of the exact solution, **b**) surface of the approximate solution, and **c**) surface of the absolute error, **d**) exact vs approximate at $x=1$, and **e**) the corresponding absolute error at $x=1$

Here, we investigate the behavior graphical representations of the approximated solution of YTADM in Fig. 7, 8. The approximate solution presented in Fig. 7a and b for various values of fractional order $\alpha \left(\alpha =0.75, 0.85, 0.95, 1\right)$ is decreases as the fractional order $\alpha$ increases. From the comparison in Fig. 8, we conclude that the obtained results of YTADM have a nice relationship with the exact solution. We have also compared the obtained results of YTADM with the results taken in [66] and the exact solution in Table 4.

**Table 4:** Approximate and comparison solutions of YTADM with the exact solution for Example 4.4

$t$	$x$	Exact solution	Approx. at $\alpha =0.9$	Approx. at $\alpha =1$	Absolute error $\alpha =1$		
					YTADM	HPTM and ADM	VIM
0.2	0.25	$0.076337$	$0.084786$	$0.076333$	$4.3390{\times 10}^{-6}$	$3.3{\times 10}^{-5}$	$3.7{\times 10}^{-5}$
	0.5	$0.305350$	$0.339146$	$0.305333$	$1.7356{\times 10}^{-5}$	$3.2{\times 10}^{-5}$	$5.0{\times 10}^{-5}$
	0.75	$0.687039$	$0.763079$	$0.687000$	$3.9051{\times 10}^{-5}$	${3.0\times 10}^{-6}$	$3.8{\times 10}^{-5}$
	1.0	$1.221402$	$1.356586$	$1.221333$	$6.9424{\times 10}^{-5}$	$1.7{\times 10}^{-4}$	$0.00$
$0.4$	$0.25$	$0.093239$	$0.107156$	$0.093166$	$7.2376{\times 10}^{-5}$	$1.17{\times 10}^{-4}$	$5.00{\times 10}^{-5}$
	$0.5$	$0.372956$	$0.428627$	$0.372666$	$2.8950{\times 10}^{-4}$	$2.67{\times 10}^{-4}$	$4.00{\times 10}^{-4}$
	$0.75$	$0.839151$	$0.964411$	$0.838500$	$6.5139{\times 10}^{-4}$	$7.5{\times 10}^{-4}$	$7.50{\times 10}^{-4}$
	$1.0$	$1.491824$	$1.714509$	$1.490666$	$1.1580{\times 10}^{-3}$	$1.27{\times 10}^{-3}$	$1.40{\times 10}^{-3}$
$0.6$	$0.25$	$0.113882$	$0.81217358$	$0.113500$	$3.8242{\times 10}^{-4}$	$5.94{\times 10}^{-4}$	$7.93{\times 10}^{-4}$
	$0.5$	$0.455529$	$1.08280575$	$0.454000$	$1.5297{\times 10}^{-3}$	$2.33{\times 10}^{-3}$	$3.00{\times 10}^{-3}$
	$0.75$	$1.024941$	$1.4140733$	$1.021500$	$3.4418{\times 10}^{-3}$	$5.30{\times 10}^{-3}$	$6.70{\times 10}^{-3}$
	$1.0$	$1.822118$	$1.8211000$	$1.816000$	$6.1188{\times 10}^{-3}$	$9.53{\times 10}^{-3}$	$1.18{\times 10}^{-2}$

Table 4 shows the exact solution, the approximate solution of YTADM for fractional order $\alpha =0.9$ and $1$, and a comparison of the exact solution and the approximate solution obtained by HPTM, ADM, and VIM found in the reference [66] for $\alpha =1$. The approximate solution found by YTADM is generally in considerably better agreement with the exact result than the numerical approach, in [66] as can be seen from the Table 4. Additionally, it is evident from the table that we get more accurate result as $\alpha \to 1$, which indicates that the other approximate solutions are likely to be valid.

## Conclusion

In this manuscript, we investigate the numerical solution of NTFPDEs using YTADM. The procedure is understandable to the readers because it consists of the direct implementation of the YT on the portion containing the fractional derivative of the given problem to change it into the algebraic form. Finally, the ADM is applied to decompose the nonlinear portion and provides a series solution to the given problem. We offered the recommended method's stability and convergence criteria along with its proof. The theoretical explanation of the suggested strategy was supported by the presentation of four illustrative instances. The findings presented in terms of figures confirm that the results obtained by the present method are in good agreement with the exact solutions for especial case $\alpha =1$ and the proposed approach gives better solution with error much near to zero. It is noted that the behavior of the approximate solution values at various $\alpha$'s is identical to that of the values obtained with the exact solution, where $\alpha =1$. This demonstrates that the approximate solution is efficient. Tables 1, 2, 3 and 4 illustrate the findings, which indicate that the suggested YTADM approach outperforms the numerical methods that have been examined and published in the literature. Consequently, we draw the conclusion that YTADM is highly effective and potent in locating numerical solutions for a broad range of NTFPDEs. Only time fractional non-linear PDEs in one-dimensional spaces were used in the current work. This may be extended to the space–time fractional PDEs with multi-dimensional spaces as well as the space fractional PDEs. Furthermore, for the purpose of addressing non-linear real-world problems, the fractional order derivative is taken in the Caputo meanings. This may also be extended to other fractional derivatives such as Caputo-Fabrizio, conformable, and Atangana-Baleanu (ABC) fractional derivative orders.

## Data Availability

No datasets were generated or analysed during the current study.
